# Deliberating the scientific evidence base for influenza transmission to raw milk consumers

**DOI:** 10.1111/risa.70077

**Published:** 2025-07-15

**Authors:** Margaret E. Coleman

**Affiliations:** ^1^ Coleman Scientific Consulting Groton New York USA

**Keywords:** risk amplification, risk perception, route and host extrapolations

## Abstract

Transmission of influenza A H5N1, commonly known as avian influenza or bird flu, from wild birds to cows on 1073 large US dairy farms in 17 states, and from cows to 41 dairy workers in five states, has raised concerns about limited evidence for transmission routes. Factors other than scientific evidence, particularly psychological, social, cultural, and political factors influencing different worldviews, support highly polarized risk perceptions about H5N1 in dairy cows, workers, and consumers. Of particular concern is the lack of scientific evidence to support federal warnings about the hypothesis that influenza transmits by the oral route to raw milk consumers. This review focuses on experimental evidence of disease transmission from 44 H5N1 inoculation studies conducted in primates, ferrets, cows, mice, cats, and dogs. Serious errors in extrapolation are apparent in the treatment of evidence for H5N1 in the media and some journal papers that unintentionally or intentionally amplify risk. Considerations of knowledge gaps and formal methods to bridge the gaps are introduced to motivate future risk analysis and facilitate building a coherent basis of knowledge to support development of rigorous evidence‐based policies and risk messaging for H5N1.

## 1 CONTEXT FOR CONSIDERING DISEASE TRANSMISSION

Transmission of influenza A is well‐characterized by models of respiratory system interactions, targeting viral–host interactions in lung cells of bronchioles and alveoli, as well as cells in corneal and ocular conjunctive tissues, and less frequently cells in the nasal mucosa (Nicas & Jones, 2009). Four possible transmission routes in humans are recognized for respiratory viruses including influenza A H5N1 (Leung, 2021; Nicas & Jones, 2009; Thangavel & Bouvier, 2014): (i) direct physical contact with infected individual (e.g., transmitting infected material via contact of particulates or fluids with face, eyes, and nose or inhalation); (ii) indirect contact (e.g., hand to fomite to face, in the absence of direct contact and inhalation); (iii) inhalation of large droplets (e.g., generated by coughing and sneezing); and (iv) inhalation of fine aerosols or sprays onto facial membranes. It is difficult to rule out transmission by multiple routes independently and simultaneously in natural transmissions to humans and other animals, though bridging studies from animal model systems (Pulit‐Penaloza et al., 2024; Zeng et al., 2019) may be helpful in drawing inferences and extrapolating between host species.

The nature and structure of influenza A viruses differ markedly from orally transmissible enteric viruses that infect and replicate in gastrointestinal (GI) tract cells and cause intestinal flu (Bosch et al., 2018; Lockhart et al., 2022; Rosenke et al., 2024). All known enteric viruses are composed of RNA or DNA surrounded by a protein coat, with structure termed “nonenveloped” (Lockhart et al., 2022). In contrast, the structure of influenza A viruses is “enveloped”; a lipid membrane “envelopes” viral RNA. Destruction of the lipid envelope by many physical, chemical, immune, and cellular barriers of mammalian GI tracts is likely to inactive influenza viruses, limiting survival, infectivity, and replication in gut tissues (Bosch et al., 2018; Lockhart et al., 2022; Weis & te Velthuis, 2021). Influenza A viruses, including avian influenza viruses, are readily inactivated by ultraviolet light, detergents, disinfectants, and heat (FDA, 2024a; Frymus et al., 2021).

Mutations and reassortments of influenza A virus strains are common, contributing to the need for updating protections for circulating strains (Awadalla et al., 2023; Nurmi, 2021; van de Ven et al., 2022). The recombination of a Eurasian ancestral H5N1 highly pathogenic avian influenza virus in 1996 from a goose in China with a North American low pathogenic avian influenza virus in 2024 created a novel genotype of H5N1 (termed clade 2.3.4.4b) associated with mild illness in US dairy cattle and dairy workers (Fillaire & Herfst, 2024; Harvey et al., 2023; Mostafa et al., 2024). Perceived risks associated with this novel H5N1 genotype have incited concerns that, as pointed out more than a decade ago by a prominent risk communication scholar (Slovic, 1999), are social constructs. Perceived risk, whether to cows, workers, or raw milk consumers, is inherently subjective and represents “a blending of science and judgement with important psychological, social, cultural, and political factors” (Slovic, 1999). Given that illness in cows and dairy workers are mild (Mostafa et al., 2024) and no epidemiologic evidence supports transmission to humans for the 2024 dairy outbreak via aerosol, ingestion, fomites, or person‐to‐person (CDC, 2024b), reporting on the US dairy herd outbreaks in 2024 appear sensationalized, inciting fear, worry, dread, and highly amplified concerns about another human pandemic.

Uncertainties about transmission of H5N1 within and between herds merit urgent action to control viral spread in dairy cows, given that recommended interventions to limit animal movement and enhance biosecurity (USDA APHIS, 2024b) were grossly unsuccessful at limiting H5N1 spread in herds in California in the fall of 2024. Affected Central Valley dairies (659 as of December 19) accounted for more than 75% of the 875 H5N1‐infected herds reported nationwide. The declaration of a state of emergency on December 18 was announced when dairy farms in Southern California were infected (California Governor, 2024).

In contrast to high uncertainties about transmission to dairy cows, transmission to workers occurs by the ocular route, via prolonged direct contact with infected animals as long noted for poultry workers and handlers. Mild cases of conjunctivitis are sporadic, with a total of 37 mild cases as of December 19 among 875 affected dairies (CDC, 2024b). Mild cases associated with the current H5N1 strain in US dairy workers differ markedly from the body of global cases in poultry workers commonly reported as cumulative global fatalities from 1996 to present linked with prior H5N1 strains. Prior H5N1 variants from global cases may be grossly unreliable predictors of public health impact in the United States given the notably sporadic and mild illnesses reported in humans and cows in dairy outbreaks in the United States.

Sensationalized reporting, unintentional misinformation, lack of transparency, and perhaps intentional disinformation about uncertainties for H5N1 in US dairy systems may be further eroding trust in public health and regulatory officials who warn about “theoretical” influenza risks to raw milk consumers by the oral ingestion route of transmission. The warnings in the media of a perceived risk to raw milk consumers appear to be based on the hypothesis that influenza H5N1 is transmitted by oral consumption and the view that raw milk is “inherently dangerous.” Risk communication scholars provide numerous examples where inconsistencies of current and past perceptions of disease severity and virus transmissibility influenced polarization about novel and feared infectious diseases and incited noncompliance with recommendations to decrease perceived risks (Balog‐Way et al., 2020; Slovic, 1999), also relevant to the 2024 H5N1 dairy outbreaks.

Guidance and warnings by US public health and regulatory officials to raw milk consumers (USDA APHIS, 2024a) were widely cited in media sources after confirmation of the initial human case of conjunctivitis in Texas in April of 2024. However, warnings about oral transmission of influenza to raw milk consumers triggered surprising responses by US consumers: surges in retail raw milk sales despite the federal warnings (Aleccia, 2024; Doan, 2024; Flynn, 2024; Kernodle, 2024a, 2024b; Leake, 2024; Makis, 2024; Tin, 2024). Media sources cited NielsenIQ figures of 25%–61% increases in retail raw milk weekly sales compared to 2023 periods (Aleccia, 2024; Lyubomirova, 2024).

**FIGURE 1:**
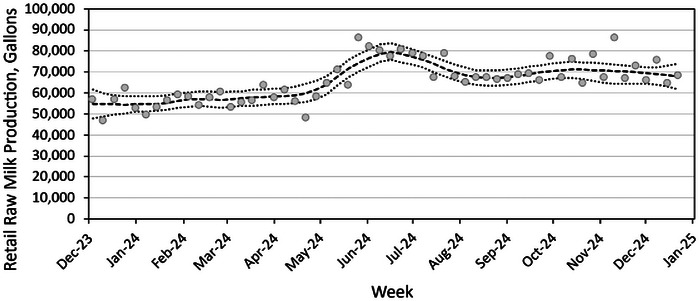
Weekly retail raw milk production from one California dairy in 2024 (Aaron McAfee, personal communication, December 27, 2024). Heavy dashed line reflects Locally Weighted Scatterplot Smoothing (LOESS) with bootstrapped 95% confidence intervals represented by dotted lines, as previously described (Stephenson et al., 2024). Note that after bulk tank milk tests were presumptive‐positive for H5N1 nucleic acid, milk from 27 November onward was diverted from the retail raw milk market to pasteurization as per policies for quarantine.

A second source of data suggesting mistrust of official warnings is weekly retail raw milk production figures for 2024 from a licensed California raw milk dairy documenting substantial and sustained increases in retail production beginning in May (Figure 1) and a strongly increasing trend for 2019–2024 (Figure S1) (Aaron McAfee, personal communication, December 27, 2024). Weekly testing of bulk milk tanks for H5N1 at this dairy was negative from April through the first week of November even though by this time in the outbreak, 279 neighboring dairies in California's Central Valley were testing positive for the virus (USDA APHIS, 2024b).

Figure 1 also conveys the magnitude and consistency of retail raw milk consumption in California, peaking at 86,000 gallons per week. If each gallon of retail raw milk purchased is consumed by a family of four, up to 344,000 Californians may regularly consume raw milk. Most US states allow access to raw milk, including 14 allowing retail access (AK, AZ, CA, CT, ID, ME, NH, NM, OR, PA, SC, WA, WY, and UT; https://www.realmilk.com/real‐milk‐legal‐map/). A recent government survey suggests that nearly 15 million people may consume raw milk in the United States (Lando et al., 2022). Despite a large number of consumers, no human cases of H5N1 have been associated with consuming raw milk at a time during which many dairies, particularly in California, reported infected cows and presumptive‐positive milk. Though absence of reported illness does not equate with absence of risk, epidemiologic evidence is a crucial component of the framework for assessing influenza transmission routes (Killingley & Nguyen‐Van‐Tam, 2013).

Errors in risk messaging about avian influenza H5N1 were pointed out as early as 2006 (Sandman, 2009; Sandman & Landard, 2006). Risk messaging errors included the need for officials to: (i) disentangle H5N1 from pandemic and seasonal influenza (H1 and H3 classes); (ii) address evidence for global, national, and regional trends using logical and robust statistical approaches; (iii) clarify the basis of evidence and inferences; and (iv) address mistrust. Similar errors appear to be at play in federal warnings alarming state and local officials, producers, and consumers about raw milk consumption (Food Standards Agency et al., 2023; Foster, 2024; Prater, 2024). Other risk communication scholars (Klemm et al., 2016) raised concerns about hype and overdramatization of swine flu in media sources when in retrospect, swine flu was not actually a human pandemic threat. The relevance of these studies to current messaging about avian flu that has caused mild illness in US cows and workers (Mostafa et al., 2024) motivates this review of available evidence for disease transmission.

Surges in US raw milk consumption despite warnings suggests mistrust of public health and regulatory officials and bias of risk messaging and policies that do not appear to be based on available scientific evidence. On the other hand, public health officials suggest that raw milk consumers could theoretically develop influenza H5N1 by ingesting or inhaling raw milk, and depicting consumers who choose raw milk as ignorant and misinformed of benefits and risks of consumption of raw milk and other foods (California Dept Public Health, 2024).

To date, human pandemic influenza has not been associated with H5N1 or any H5 viruses, but with other influenza A virusus with distinct characteristics, primarily efficient replication, efficient transmission person‐to‐person or by aerosols, and binding with high affinity to human receptors; pandemics include H1N1 in 1918 and 2009, H2N2 in 1957, and H3N2 in 1968 (Graziosi et al., 2024; Guo et al., 2024; Herfst et al., 2017). Thus, public health officials typically consider influenza viruses to demonstrate high pandemic potential when the virus gains the ability to transmit person‐to‐person or by aerosols (Guo et al., 2024), conditions more likely to cause widespread serious illness than by the oral transmission route. Various studies of engineered viruses documented combinations or accumulations of multiple genetic substitutions were needed to engineer an H5N1 hybrid virus that transmitted ferret‐to‐ferret (Linster et al., 2014). No such variants have occurred naturally by mutation and reassortment in nearly three decades since documentation of the first rare sporadic human cases via direct exposure to sick, dying, or dead birds (Graziosi et al., 2024). Furthermore, influenza H7N9 strains permitting transmission between ferrets appear insufficient to cause widespread outbreaks in humans (Braun et al., 2023), pointing to the need for bridging data to extrapolate from ferrets to humans.

The next section of this perspective focuses on one of multiple categories in the framework for determining routes of influenza transmission (Killingley & Nguyen‐Van‐Tam, 2013): experimental evidence on H5N1 transmission from 44 inoculation studies (6 in vitro and 38 in vivo). Other categories in the framework for assessing influenza transmission are introduced in Section 3. The focus on evidence of influenza transmission is warranted for future deliberations regarding evidence supporting and attenuating the hypothesis of oral transmission of influenza to raw milk consumers. For brevity in this perspective, highlights from the inoculation studies are provided in the following section, and tables on disease transmission for the 44 studies (Tables S1–S6) and additional details on the studies are provided in the Supporting Information to inform future microbial risk assessment.

The purpose of this perspective is to facilitate building a coherent basis of knowledge to support rigorous, evidence‐based risk analysis for avian influenza H5N1. Open deliberation of this rich base of evidence is essential to future extrapolations for robust and transparent risk analysis for H5N1 in dairy consumers. Notably, the need to turn or return to fundamentals of the scientific method and objective evaluation and analysis was emphasized in 2024 books by two physician‐scientists, Francis Collins (*The Road to Wisdom: On Truth, Science, Faith, and Trust*) and Marty Makary (*Blind Spots: When Medicine Gets It Wrong, and What It Means for Our Health*). These authors also point us to contemporary and foundational aspects of human nature that have hampered effective risk analysis in our society by the illusion that we humans are purely rational objective creatures without emotional influences that drive our beliefs and biases. The intent of this perspective is to continue the work of Drs. Collins and Makary and rebuild trust between stakeholders and public health and regulatory officials.

## 2 DOSES CAUSING NO DISEASE TRANSMISSION AND ILLNESS

H5N1 virus has not been tested in human volunteer studies to date. Human data and models exist for attenuated human seasonal influenza A strains (H1N1 and H3N2; vaccine strains) administered to human volunteers (Nicas & Jones, 2009). Other dose–response models incorporated data for intranasal inoculation of volunteers with H3N3 (five dose groups), as well as the data from the previous study with H1N1 (four dose groups) and with H3N2 (nine dose groups) (QMRAwiki Influenza Dose Response for Intranasal). Models for influenza transmission routes in humans are available (Leung, 2021; Nicas & Jones, 2009; Thangavel & Bouvier, 2014).

Experimental evidence from in vitro cell assays can provide relevant information for assessing infectivity and virulence, but not about transmission. Human in vitro data from H5N1 inoculation studies exist for lung cancer cells (Pulit‐Penaloza et al., 2024), differentiated nasal epithelial cells (Richard et al., 2020), differentiated alveolar cells and macrophages (Yu et al., 2011), colorectal tissues (Shu et al., 2010), and retinal pigment epithelial cells (Michaelis et al., 2009). In addition, in vitro data exist from differentiated alveolar cells of ferrets (Zeng et al., 2019). A relevant finding from in vitro experiments is the demonstration that the lower temperature of the human upper respiratory tract may contribute to ineffective transmission of H5N1 in humans, even though viral attachment to receptors occurs (Pulit‐Penaloza et al., 2024). However, these in vitro cellular systems are grossly oversimplified; they lack the complexity of interactions between respiratory, immune, gut, neural, and vascular systems, and exclude natural microbiota and their metabolites, that together modulate host–pathogen interactions and contribute to health and homeostasis (Huang et al., 2023; Manos, 2022; Marrella et al., 2024).

The large body of in vivo evidence, including 38 H5N1 inoculation studies described in this section and in Tables S2–S6, merits careful scrutiny in assessing what data might be applicable for making sound scientific inferences about the likelihood and severity of human illness following direct contact, ocular, oral, aerosol, or fomite exposures to H5N1. As demonstrated for other pathogens including biothreat agents, similar bodies of evidence from animal inoculation studies supported identification of the doses associated with no disease transmission and mild, moderate, and severe illness to select appropriate exposure guidelines to protect human health (McClellan et al., 2018; Watson et al., 2018). Highlights of the body of in vivo evidence for H5N1 are presented below.

The outcomes of experimental, and natural, pathogen transmissions are dependent on many factors (Coleman et al., 2018; Leung, 2021; Lunn et al., 2019; Rodriguez et al., 2017). Successful transmission depends primarily on infectivity and virulence of the virus strain, viral load transferred, and environmental stresses on the virus before and after exposure, likely differing by transmission route (Leung, 2021).

A cohesive and coherent body of knowledge characterizing dose–response relationships for humans is key to estimating risk and designing management strategies to reduce risk or to reduce severe risk. The most influential parameters identified for influenza in a recent review of respiratory studies supporting quantitative microbial risk assessment (QMRA) and decision‐making were: (i) viral concentration or dose; and (ii) viral surface transfer efficiency (Tang et al., 2024). Although dose–response data for humans and other mammals are sparse, Tang and colleagues (2024) note utility of comparing relative importance of potential transmission pathways, such as inhalation of aerosol droplets or contact transmission, as well as definition of a practical acceptable disease burden.

In addition, experimental data on viral doses causing the full spectrum of human disease (asymptomatic, mild, severe, and fatal disease) are essential to selecting appropriate animal models for extrapolation to predict dose–response relationships and outcomes for humans. Both nonhuman primates (NHPs) and ferret models can present outcomes similar to human disease from previous H5N1 strains in poultry workers (pneumonia, acute respiratory distress syndrome, and respiratory failure) (Tang et al., 2024), and thus may generate relevant data for extrapolation to human risk analysis.

### 2.1 Doses causing no disease transmission

One NHP study (Rosenke et al., 2024) reported no disease transmission by administration of a single very high H5N1 dose (10 million times the dose infecting 50% tissue culture cells) from the mouth to the stomach (orogastric inoculation). See Table S2 and the Supporting Information for more information on this study.

One ferret study (Lipatov et al., 2009) reported no disease transmission by oral administration of a single very high H5N1 dose in liquid medium (1 million times the dose infecting 50% of injected embryonated eggs). See Table S3 and the Supporting Information for more information on this study.

One mouse study (Eisfeld et al., 2024) reported no disease transmission by the lowest of a four‐dose series inoculating ∼100 viral counts (plaque forming units or pfu) by nose and throat. In an earlier mouse study (Lipatov et al., 2009), one of three H5N1 strains inoculated in the stomach at a high dose (approximately 1000 times the dose infecting 50% of injected embryonated eggs) caused no disease transmission. See Table S5 and the Supporting Information for more information on this study.

One cat study (Vahlenkamp et al., 2008) reported no disease transmission at the lowest three of four doses (1, 100, and 10,000 times dose infecting 50% of injected embryonated eggs) by multiple routes (ocular, nasal, and oral pharyngeal). See Table S6 and the Supporting Information for more information on this study.

### 2.2 Doses causing illness

For 10 NHP studies, very high H5N1 doses caused illness when administered to the nose (intranasal), the throat (intratrachael), and combined routes including nose, throat, and eye (ocular) or via aerosol. See Table S2 and the Supporting Information for more information on these studies.

For nine ferret studies, H5N1 administration to the nose (intranasal), the throat (intratrachael), eye (ocular), oral feeding of inoculated meat, and injection in the stomach (intragastric) in minced meat caused illness. Most doses were high, exceeding 1000 viral counts (pfu) or 10,000 times the dose infecting 50% of tissue cultured cells or injected embryonated eggs. See Table S3 and the Supporting Information for more information on these studies.

For cows, two studies (Baker et al., 2025; Kalthoff et al., 2008), inoculating at very high doses (a million times the dose infecting 50% of tissue cultured cells or 10^8.5^× the dose infecting 50% of injected embryonated eggs) causing caused mild illness. See Table S4 and the Supporting Information for more information on these studies.

For seven mouse studies, H5N1 administration to the nose (intranasal), the throat (intratracheal), nose and throat (nasopharyngeal), injection in the stomach (intragastric), or whole body exposure to aerosol caused illness. Most doses were high, exceeding 1000 viral counts (pfu) or 10,000 times the dose infecting 50% of injected embryonated eggs. See Table S5 and the Supporting Information for more information on these studies.

For five studies inoculating cats or dogs (Giese et al., 2008; Reperant et al., 2012; Rimmelzwann et al., 2006; and Vahlenkamp et al., 2008, 2010), H5N1 administration at very high doses (>10,000 times the dose infecting 50% of tissue culture cells or injected embryonated eggs) to the throat (intratrachael), eye, nose, and throat (oculo‐nasopharyngeal), injection in a vein (intravenous) or the stomach (intragastric), or oral feeding caused illness. See Table S6 and the Supporting Information for more information on these studies.

### 2.3 Studies for indirect exposures

In addition to direct inoculation studies noted in Subsections 2.1 and 2.2, experimental scenarios were tested for indirect exposures of inoculated animals paired with naïve animals or exposed to contaminated surfaces (fomites) in: seven ferret inoculation studies (Belser et al., 2012, 2024; Eisfeld et al., 2024; Maemura et al., 2023; Pulit‐Penaloza et al., 2024; Restori et al., 2024; Richard et al., 2020); a mouse study (Eisfeld et al., 2024); and dog and cat studies (Giese et al., 2008; Rimmelzwann et al., 2006). Results for transmission by these indirect scenarios are summarized in the Supporting Information.

### 2.4 Summary of available evidence relevant for extrapolation to humans

The body of experimental evidence described in this section merits careful scrutiny in assessing what data might be applicable for making sound scientific inferences about the likelihood and severity of influenza transmission to human exposed to H5N1 by multiple routes.

For primates, the experimental data from human in vitro cell assays (human lung epithelial cells, alveolar macrophages, and other tissue cultured cells), have contributed to the body of knowledge for influenza A including H5N1 but provide no evidence for disease transmission. Overall, in vitro and ex vivo studies in human cells provide no evidence for transmission or distribution (tropism) to target organs, though infectivity and replication in respiratory and colonic cells is demonstrated. Thus, results for replication in ex vivo colonic tissue are not definitive evidence for disease transmission by the oral route, consistent with Mims’ observation (Mims, 1989) that susceptible host cells in vitro and ex vivo can escape natural infection in vivo when anatomical, physiological, chemical, and cellular conditions provide sufficient trophic barriers.

For 10 NHP studies, all provide evidence for respiratory transmission and mechanisms consistent with human pathogenesis. The lack of clinical disease from oral (orogastric) inoculation as the sole inoculation route (Rosenke et al., 2024) and in combined routes (Baskin et al., 2009; Cillóniz et al., 2009; Mooij et al., 2021; Muramoto et al., 2014; Rimmelzwaan et al., 2001; Watanabe et al., 2018) warrant further consideration for extrapolation to test the hypothesis that raw milk consumers may acquire influenza via oral transmission.

Similarly, evidence of ineffective transmission of H5N1 via the oral ingestion route in ferrets (Bertran & Swayne, 2014; Lipatov et al., 2009) and the necessity of inoculating oral doses orders of magnitude higher than intranasal doses for transmission in ferrets via respiratory and systemic disease (Bertran et al., 2014; Lipatov et al., 2009) merit consideration for hypothesis testing regarding oral transmission of influenza in humans.

Three research groups inoculated both ferrets and mice intranasally (Eisfeld et al., 2024; Lipatov et al., 2009; Maemura et al., 2023), and mice appear exquisitely more susceptible to H5N1 than ferrets. Lethal dose estimates for mice inoculated by the intranasal route (LD_50,IN_) ranged from 2.2 to 48 pfu for three H5N1 strains, while a single high dose of 10^6^ pfu to ferrets was lethal for all three animals for each H5N1 strain tested (LD_50_ for ferrets not estimated) (Maemura et al., 2023). Another study reported a comparable LD_50,IN_ of 31.6 pfu in mice inoculated with the cow‐H5N1 strain (Eisfeld et al., 2024). Another mouse study reported high infectivity (ID_50,_ dose causing 50% infection) and lethality (LD_50_) by intranasal and aerosol inoculation routes (Belser et al., 2015): infectivities of 8.9 pfu ID_50,AE_; 15.8 pfu ID_50,IN_ and lethalities of 16 pfu LD_50,AE_ and 89 pfu LD_50,IN_). The very high infectivity and mortality of the mouse model may have limited representativeness for extrapolating to humans.

Although mouse inoculation studies may be of limited value for extrapolation to human transmission, mouse studies on mechanisms of infection and resistance may be relevant. For example, pathogenicity was consistently observed in mouse respiratory or systemic infections but was variable by intragastric inoculation, with lack of definitive determination of infection and replication of GI tissues and cells (Lipatov et al., 2009).

Some significant limitations for the body of experimental inoculation studies include the dose units relative to egg or tissue culture effect rather than more direct coun (pfu). Without expression of the administered doses in the appropriate units as actual counts or pfu, comparisons between studies are problematic, as is extrapolation to potential human doses and responses. Furthermore, excellent reviews document many limitations and strengths of use of different animals models as surrogate hosts for predicting outcomes of viral exposures to humans (Chan et al., 2013; Imai et al., 2013; Leung, 2021; Lunn et al., 2019; Nguyen et al., 2021; Thangavel & Bouvier, 2014).

#### 2.4.1 Mechanistic evidence for barriers to infection and promotors of recovery

Mechanistic information on repair and recovery of murine influenza depicts immunomodulating and antiviral activity present in raw milk (see Table S7 for studies characterizing 16 antiviral components), including lactoferrin, the milk microbiome, and a variety of enzymes and peptides. The globular glycoprotein lactoferrin reduced or repaired influenza‐induced inflammatory pathology in mouse lung and colon tissues via the gut‐lung axis, the complex interactions between the mucosal immune system, the gut microbiota, inflammatory factors, and the intestinal barrier (Huang et al., 2023). Signs of influenza in mice inoculated with H5N1 (weight loss, hair discoloration, lung damage, inflammatory cell infiltration, vascular dilation and congestion, damage of intestinal barrier, and diarrhea and unformed feces) improved with lactoferrin treatment via multiple mechanisms including: inhibiting inflammation; regulating gut microbiota homeostasis; promoting colonization resistance by increasing the abundance of beneficial bacteria and decreasing pathogenic bacteria; and repairing damaged intestinal and respiratory mucosal barriers (Huang et al., 2023).

In addition, intestinal damage in mice following primary respiratory infection via intranasal inoculation (Huang et al., 2023) was associated with indirect immune‐mediated intestinal immune damage and disruption of microbiota, not GI infection and replication. Connections between mucosal tissues in the gut and respiratory tract modulate pathology along the gut‐lung axis in influenza A H5N1 and other respiratory disease including asthma, chronic obstructive pulmonary disease, COVID‐19, influenza, and pneumonia (Enaud et al., 2020; Ou et al., 2023; Y.‐H. Wang et al., 2023). For example, the dietary immunomodulator lactoferrin reduced H5N1 lung and intestinal injury, alleviated inflammation, rebalanced gut microbiota, and restored integrity of intestinal wall and lung tissue (Huang et al., 2023).

For dairy farm workers, the available observational data on transmission pathways for influenza A H5N1 are consistent with ocular exposure via direct physical contact with infected cows (CDC Newsroom, 2024). For humans contacting avian or fur‐bearing farmed animals, direct contact with infected wild or farmed animals may involve both direct contact and inhalation of large droplets or fine aerosols over short range (Szablewski et al., 2023; Zhao et al., 2023). It is unclear if indirect contact with fomites or aerosols in the absence of contact with an infected animal contributes to human disease for dairy or poultry workers or bovine transmission within and between herds. Uncertainty also exists regarding transmission within and between dairy herds.

In addition, failure of H5N1 to transmit in ferrets via aerosols (Belser et al., 2012; Eisfeld et al., 2024; Maemura et al., 2023; Pulit‐Penaloza et al., 2024; Restori et al., 2024; Richard et al., 2020) or contact with fomites (Pulit‐Penaloza et al., 2024) are consistent with direct contact and ocular exposures as primary routes for transmission to humans from recent epidemiologic investigations (CDC Newsroom, 2024). Overall, evidence of transmission from inoculation and observational studies is consistent with a low pandemic potential of H5N1 for human influenza based on the 2024 dairy outbreaks.

## 3 FUTURE DELIBERATIONS FOR QUALITY RISK ANALYSIS

The need is urgent for robust transparent risk analysis processes based on coherent bodies of evidence for avian influenza H5N1 transmission that meet analytical quality criteria, such as those developed by the Applied Risk Management specialty group of the Society for Risk Analysis (Lathrop et al., 2024; Society for Risk Analysis, 2020; Waller et al., 2024). Improvements in clairity and transparency about the scientific evidence, or the lack of it, for H5N1 transmission routes are essential for quality analysis and appropriate risk messaging to counter widespread misinformation or disinformation in various media and to inform decision‐making, particularly about human consumption of raw milk from healthy dairy cows and transmission within and between herds.

### 3.1 Applying the evidence in the framework for influenza transmission

Considering the framework for influenza transmission (Killingley & Nguyen‐Van‐Tam, 2013), the following evidence merits wide deliberation to assess the hypothesis that influenza transmits by the oral route to humans and develop evidence‐based strategies for risk communication and risk management.

#### 3.1.1 Inoculation studies

Of 44 inoculation studies reviewed herein, the most relevant anatomically, immunologically, and physiologically for extrapolation to humans reported no disease transmission for NHPs inoculated with a high dose of H5N1 by the orogastric route (Rosenke et al., 2024). The same high H5N1 dose inoculated intranasally caused mild illness and intratracheally caused severe and fatal illness in NHPs. The finding of no influenza transmission to NHPs inoculated with high oral dose warrants deliberation for extrapolation to oral transmission to humans, particularly in light of additional barriers to infection and replication in the GI tract as introduced subsequently.

Experimental evidence from the 44 inoculation studies with H5N1 in mammals (Tables S2–S6) will likely be biased predictors for assessing risk to human raw milk consumers without targeted research and mechanistic data for robust interspecies extrapolations.

Various strategies could be useful for extrapolating from animal models and in vitro findings to predict human oral transmission. Chemical risk assessors apply formal methods to bridge knowledge gaps about transmission routes and mechanisms to increase confidence or rigor, making inferences by analogy (e.g., read‐across methodology, quantitative structure–activity relationship [QSAR] models), particularly when screening new and emerging hazards (Alexander‐White et al., 2022; Fisher et al., 2024; Lee et al., 2024; Lizarraga et al., 2023). The tularemia project documented by McClellan et al. (2018) considered read‐across by host and route for sparse human data for oral exposures and more extensive data for NHPs that improved confidence in disease transmission models.

#### 3.1.2 Epidemiologic evidence

No epidemiologic evidence documents oral transmission of H5N1 to raw milk consumers. Before a California recall in November of 2024, more than 263,000 gallons of raw milk from infected cows (∼4.6 million 240 mL servings) were already in the retail market. CDC reported that US human H5N1 cases are rare, sporadic, and mild in 2024 dairy outbreaks (CDC, 2024a, 2024b), with no uptick of influenza or influenza H5N1 in humans in that month or that year, despite potential exposure to raw milk consumers. No epidemiologic evidence of oral transmission of enveloped influenza A viruses including H5N1 has been documented in the absence of direct animal contact and respiratory or systemic infection (Minodier et al., 2015; Vahlenkamp et al., 2010).

#### 3.1.3 Modeling deposition and clearance informing biological plausibility

Models for testing mechanisms of deposition and clearance of influenza viruses in respiratory and ocular systems are well documented (Nicas & Jones, 2009; Leung, 2021; Quirouette et al., 2020; Thangavel & Bouvier, 2014). For humans exposed to infected poultry, avian influenza H5N1 was associated with rare but severe and fatal human respiratory illness, including pneumonia and acute respiratory distress syndrome. No such models exist for hypothetical oral transmission to humans.

Deliberations are needed regarding evidence of deposition and clearance of influenza in the GI tract, particularly due to its nature as an enveloped virus. Factors that differentiate transmission mechanisms typical of enveloped influenza A viruses from those characterized for enteric viruses (adenovirus, astrovirus, enterovirus, hepatovirus, norovirus, rotavirus, and sapovirus) adapted to infect the GI tract and causing intestinal flu warrant consideration regarding the hypothesis of oral transmission for H5N1 (Lockhart et al., 2022).

Multiple studies (Minodier et al., 2015; O'Brien et al., 2021; Shu et al., 2010) acknowledge that no direct evidence exists to support H5N1 passage though the gauntlet of physical, chemical, immunological, and microbial barriers in intact GI tract tissues required for infection and replication in the GI ecosystem as described even though intestinal symptoms and pathology are sometimes reported. Furthermore, multiple research teams (Froggatt & Heaton, 2022; Minodier et al., 2015) caution that detection of viral antigens in intestinal tissues and feces without confirming viability is insufficient evidence to document influenza A transmission via the oral route with infection and replication in gut cells.

Previous studies from the biothreat literature (Eigelsbach et al., 1965; Hornick et al., 1966; Kuolee et al., 2007; McClellan et al., 2018) were consistent with a distinct pattern of high infectivity for respiratory exposures (∼10 pathogen cells) and the low infectivity for oral ingestion (>10^7^ pathogen cells) of a biothreat agent across humans and laboratory animals. Mechanistic data (Adcock et al., 2014; Blume et al., 2016) provide supporting information on the influence of GI and respiratory system barriers to tularemia infection consistent with low infectivity and virulence by the oral route with minimal replication in gut mucosal cells. This pattern of high infectivity for respiratory and low infectivity for oral exposures to tularemia may reflect the gauntlets of barriers that distinguish respiratory and GI tracts, barriers that may also affect H5N1 transmission.

Future research modeling the progression of “key events” or stages in pathogenesis for oral transmission pathways that drive infection processes toward illness or repair and recovery would be relevant to testing the hypotheses for oral transmission of H5N1 in humans (Abe et al., 2021; Buchanan et al., 2009; Coleman & Marks, 2000; Coleman et al., 2017, 2018; Julien et al., 2009). “Key events” could be modeled for each stage of the hypothesized ocular, oral, and respiratory pathways for H5N1, predicting outcomes of interactions of virus with host target cells incorporating data for variables representing local and systemic host defenses, resident microbiota, and therapeutics that might limit or prevent viral survival, attachment, infection, and replication of H5N1 or maximize its clearance (AbuBakar et al., 2023; Koutsakos et al., 2019; Quirouette et al., 2020; Sachak‐Patwa et al., 2023).

Future use of simulation software and experimental systems may provide additional confidence in assessing oral transmission of H5N1. *In* silico tools are available for modeling deposition and clearance of particles and pathogens in lungs (Asgharian et al., 2012; Lazaridis, 2023; Lejon, 2019; Pöhlker et al., 2023) and GI tract interactions (Marzorati, 2018; Marzorati et al., 2021; Meddah et al., 2001; Verhoeckx et al., 2015; Q.‐Y. Wang et al., 2011; X. Wang et al., 2024; Zhang et al., 2022) of humans and other animals. Another in silico model compared H5N1 and H1N1 and differences in host target cell binding affinity (cell tropism) that appear to contribute to differences in severity (Dobrovolny et al., 2010). Similarly, in silico models describe within‐host influenza infection dynamics that account for differing outcomes linked to various immune system responses (Sachak‐Patwa et al., 2023). Such simulation models may be useful in testing feasibility, kinetics and dynamics of transmission, and progression, treatment, and prevention of infections by ocular, oral, and respiratory routes.

Computational models of the human respiratory tract incorporate advanced understanding about the dynamics of influenza deposition and clearance via advection, likely a relevant process for H5N1 that would be entrained upward with pericilliary fluid toward the nose and mouth to decrease influenza persistence in the respiratory tract, thus likely decreasing the likelihood and severity of infection (Quirouette et al., 2020). Furthermore, computational models have reproduced influenza H1N1 infection dynamics in mice and distinguished between hosts of different immune competence (Sachak‐Patwa et al., 2023).

Other relevant advances document protective microbiota in the respiratory and GI tracts that communicate along the gut‐lung axis, interfere with pathogen attachment, invastion and replication, as well as enhance clearance and tissue repair (Enaud et al., 2020; Marrella et al., 2024; Ou et al., 2023; Y.‐H. Wang et al., 2023). Potential exists for enhancing gut‐lung axis protections and innate defenses against H5N1 transmission through dietary and therapeutic or preventative supplementation with probiotics linked with reduction in the likelihood or severity of influenza A including H5N1 (Enaud et al., 2020; Y.‐H. Wang et al., 2023), and reductions in secondary bacterial pneumonia often associated with severe influenza (Liang, 2023).

Furthermore, combinations of experimental and in silico systems may also be helpful to verify and extend results from oral exposures of high doses of H5N1 in laboratory animals. For example, given that orogastric delivery of a high density of virions to upper GI tissues in NHPs caused no clinical disease (Rosenke et al., 2024), collaborations between experimental and in silico researchers could test hypotheses about effects of low and high local doses on overcoming immune protections for animal cells directly contacting the viral inoculum. It is possible that natural oral H5N1 exposures may prove ineffective for transmission of clinical disease in humans due to multiple levels of trophic barriers.

The extent of inactivation of H5N1 and other enveloped viruses by physical, chemical, immunological, and microbial defenses active in the human mouth, stomach, and small and large intestines could be tested using in silico computational models. Recent development of 3‐D models of human intestine that include a simplified human gut microbiota in addition to human gut and immune cells (Moysidou et al., 2024) may support stronger inferences about H5N1 as a potential foodborne pathogen using a system more representative of the human gut ecosystem than oral inoculation studies documented in herein.

#### 3.1.4 Modeling to quantify routes of transmission

Mathematical models of respiratory exposures are available for influenza (Nicas & Jones, 2009; Leung, 2021; Thangavel & Bouvier, 2014), but no such models quantifying oral transmission of H5N1 are available to date.

### 3.2 Considerations for future risk assessment

Formal methods of inferencing are required for assessing risks of H5N1 by ocular, oral, and respiratory routes, as well as across host species. Particularly for the oral route, robust inferences based on available evidence are needed to reduce existing uncertainty about the potential for H5N1 to transmit via the oral route (e.g., via raw milk, poultry meat, and eggs) and cause pandemic influenza with widespread serious human illness.

Previous studies had reported that H5N1 was not considered a foodborne pathogen for poultry in 2010, even though H5N1 may be present in foods (Bauer, 2010). Others more recently conducted a qualitative risk assessment and reported H5N1 as a negligible foodborne risk, again focused on poultry (Food Standards Agency et al., 2023). Applying the qualitative scales used in this UK FSA assessment, the lack of evidence for human cases by oral transmission suggests negligible to very low occurrence, negligible severity, and high uncertainty for US raw milk consumers. A third risk assessment study available as a preprint under peer‐ review (Koebel et al., 2024) considered data from one of the 44 inoculation studies discussed herein, a 2009 ferret study including a single very high dose (1 million times the dose infecting 50% of injected embroyonated eggs) (Lipatov et al., 2009), apparently considered laboratory ferrets equivalent to humans without adjustment for anatomical, immunological, microbiological, and physiological differences.

These three risk assessment studies appear inconsistent, and merit more scrutiny. Neither of the former two is consistent with warnings about consuming raw milk. The latter (Koebel et al., 2024) supports the warnings, assuming equivalence of ferret and human dose–response relationships and likely incorporating many other unverified assumptions and simplifications of convenience. The Koebel study under review (2025) estimated median probabilities of infection equivalent to ∼1/1000 risks for raw milk consumers drinking 240‐mL servings, a rate inconsistent with epidemiologic evidence of no influenza illnesses for up to 4.6 million servings of retail raw milk in California before the 2024 recall.

One great value of modeling under uncertainty is that incorporating alternative assumptions can advance understanding of the underlying biology of the system and thus enhance its management. For example, models incorporating extrapolations from both NHP and ferret studies as potential surrogates of human oral dose–response relationships for influenza could be informed by targeted research bridging NHP and ferret studies. Sharper knowledge of the system might then lead to more informative assessments of the magnitude and severity of oral influenza, and subsequently more effective interventions for protection of human and animal health. These risk assessment studies point out the need for deep deliberation by the wider microbial risk analysis community, including consideration of an alternative hypothesis that enveloped H5N1 lacks key characteristics for viral infectivity and replication in primate GI systems (Bosch et al., 2018; Lockhart et al., 2022; Rosenke et al., 2024).

Future dose–response assessment might initially begin with a hypothesis documented in 2013: the transmission scenario for humans contacting high potential H5N1 concentrations in heavily shedding poultry imply that a high threshold viral concentration was required for effective transmission to humans, and those exposed to low concentrations were at very low risk of infection (Van Kerkhove, 2013). Subsequent researchers noted a key to predicting the likelihood and severity of risk for influenza is viral dose (Lunn et al., 2019), the nexus or link between exposure and all upstream processes causing or suppressing disease in different hosts, as Lunn and colleagues note.

Regarding H5N1 doses administered to mammals in Tables S2–S6, doses lethal to both NHPs (Rosenke et al., 2024) and ferrets (Lipatov et al., 2009) by the intranasal/intratracheal route were nonpathogenic by the oral route. This pattern is consistent with high‐quality data from tularemia studies that inoculated hundreds of human volunteers, NHPs, and mice. Very high oral doses of Francisella *tularensis* (10^10^ bacteria) were required for GI and febrile illness with high‐titer response, compared to the very low median aerosol dose of 10 bacteria causing respiratory infection, febrile tularemiosis, and seroconversion in human volunteers (Hornick et al., 1966; McClellan et al., 2018).

Similarly, doses of H5N1 in aerosols or fomites from ferret experiments were insufficient for transmission to naïve animals, and even direct contact between inoculated and naïve cats and dogs was insufficient to cause cross‐species transmission (Giese et al., 2008; Pulit‐Penaloza et al., 2024).

Researchers have suggested that nonlinearities in relationships between viral dose and the likelihood and severity of responses in different hosts may provide mechanistic barriers to spillover to new hosts (Fauziah et al., 2024; Imai et al., 2013). Recent microbial risk assessment studies for respiratory pathogens (Boles et al., 2022; Hamilton et al., 2024; McClellan et al., 2018; Tang et al., 2024) point out the need for improvements: characterizing dose‐dependent outcomes; extrapolating across gaps in knowledge of administration routes and host species; and the need to involve stakeholders in decision making to build trust. Transparency about the basis of evidence and inferences from it are crucial to quality risk analysis practices that respectfully engage stakeholders and effectively manage influenza risks (Seno‐Alday, 2018; Waller et al., 2024).

### 3.3 Considerations for future risk communication

Development of evidence‐based risk communications are needed for a variety of topics and target audiences. A previous study that considered dissonances between ideals and reality for influenza H1N1 (Driedger et al., 2021) is worth consideration for H5N1 as well. A body of risk communication studies of past viral pandemics (Balog‐Way & McComas, 2022; Balog‐Way et al., 2020; Klemm et al., 2016; Q. Wang et al., 2020) might inform initiatives underway to limit spread of H5N1 in dairy herds.

While public health and regulatory officials acknowledge uncertainties about oral transmission to humans (CDC, 2024b; FDA, 2024b), improvements are necessary to increase transparency about the basis of evidence. Current risk communications appear dissonant with the lack of evidence for H5N1 oral transmission in primates (Rosenke et al., 2024) and conflicting evidence across animal models including: NHPs (Baskin et al., 2009; Cillóniz et al., 2009; Mooij et al., 2021; Muramoto et al., 2014; Rimmelzwaan et al., 2001; Rosenke et al., 2024; Watanabe et al., 2018); ferrets (Bertran & Swayne, 2014; Lipatov et al., 2009); mice (Eisfeld et al., 2024; Guan et al., 2024; Lipatov et al., 2009); and cats (Reperant et al., 2012; Rimmelzwaan et al., 2006; Vahlenkamp et al., 2010). The body of evidence merits more rigorous and transparent treatment, particularly regarding integration of scientific evidence with judgmental heuristics and cultural, ideological, political, and social factors influencing risk perceptions (Balog‐Way et al., 2020; Slovic, 1999). Furthermore, confirmation bias (Cox & Popken, 2008) for officials whose worldview that raw milk is “inherently dangerous” appears likely to be influencing messaging on H5N1 oral transmission to raw milk consumers.

Evidence of mistrust of official warnings to raw milk consumers from multiple sources (Aleccia, 2024; Lyubomirova, 2024; Aaron McAfee, personal communication, December 27, 2024) merits future risk communication work to rebuild trust (Balog‐Way et al., 2020; Sandman & Landard, 2006; Slovic, 1999). Peak weekly retail production in California was 86,183 gallons in May (compared to ∼55,000 gallons from January to April), following warnings to raw milk consumers about influenza H5N1 (Figure S1). This uptick in weekly raw milks sales continued at ∼10,000 gallons higher than the 2024 period prior to May's warnings. Despite potential exposures, CDC has reported no H5N1 disease in raw milk consumers (CDC, 2024b), notably those California consumers in November who were potentially exposed to up to 4.6 million servings of retail raw milk from infected cows. An unknown fraction of production may have been consumed prior to the recall (Gutierrez, 2024; Harvey & Tupper, 2024), with no reported illnesses. Transparency and deeper systematic analysis is required to address the many factors driving amplification of perceived risk for H5N1 specifically, and benefits and risks in general (Coleman et al., 2021; Dietert et al., 2022; Stephenson et al., 2024), for raw milk consumers.

Media dramatization and unsupported claims by scientists are issues that complicate effective risk communication about influenza transmission from livestock, poultry, and foods to humans (Klemm et al., 2016). Media coverage of H5N1 as a potential hazard to raw milk consumers documents mistrust about communications inciting fear or engaging actively in fear‐mongering (Kernodle, 2024a; Leake, 2024; Makis, 2024; Molteni, 2024; Palese & Wang, 2012) in the absence of evidence for oral transmission to humans. Future analysis by risk communication scholars of the role of the media and officials in fear‐mongering and dramatization of H5N1 as structured for swine flu (H1N1) is merited (Klemm et al., 2016).

Another need for future risk communications about H5N1 is developing guidance for diverse stakeholder groups (Burns et al., 2013), currently in the United States for dairy farm workers on appropriate use of personal protective equipment to minimize exposure to H5N1 (Bagdasarian, 2024). For example, effective protection from droplet splash during milking likely differs for workers on small farms and those on large concentrated animal feeding operations or CAFOs where tens of thousands of cows maybe be confined. In the latter, workers may be positioned below the cows during milking, whereas workers on small family farms may be positioned at the same level as cows. Also, deliberations of evidence for risk messaging regarding the perceived need for treatment of family members and other contacts of cases with antivirals or future vaccines should include risk scholars, considering that the available evidence does not support indirect transmission of H5N1 to ferrets by fomites (Pulit‐Penaloza et al., 2024).

### 3.4 Considerations for future risk management

Risk management interventions for dairy workers (USDA APHIS, 2024a) may be effective in limiting human H5N1 infections to rare sporadic cases. From June to September, no new cases in dairy workers were reported after prior cases of mild illness (conjunctivitis) in workers from Texas in April (1), Michigan in May (2), and Colorado in June (1). As of December 19, 33 sporadic mild cases of conjunctivitis were reported in dairy workers of the Central Valley among 37 to date (CDC, 2024b).

Many countries including China (Zhao et al., 2023), Egypt (Abdelwhab, 2014; Gomaa et al., 2015), and the United Kingdom (Animal & Plant Health Agency, 2023; Oliver et al., 2022) have successfully managed worker, food preparer, and consumer exposures to H5N1, and reduced numbers of cases and mortality to humans (WHO, 2024) despite continuing avian deaths around the world (European Food Safety Authority et al., 2024a, 2024b). The need for warnings about perceived risk to raw milk consumers from H5N1‐infected cows (USDA APHIS, 2024a) requires additional scrutiny to develop evidence‐based communications for dairy consumers.

Risk management interventions for dairy herds (isolation and premovement testing of cows prior to and following transport to other herds and other states; enhanced biosecurity measures) (USDA APHIS, 2024a) appear effective in limiting H5N1 spread in most states (CDC, 2024b; USDA APHIS, 2024b). From the very short interval of March 25th to April 2nd, dairy cow cases were reported in six states (Texas first, then, Kansas, Michigan, New Mexico, Ohio, and Idaho) that may or may not reflect interstate movement of infected cows (USDA APHIS, 2024b).

The current hypotheses about transmission in dairy cattle require further scrutiny to determine the local factors driving transmission in California when controls for animal movement and biosecurity appear grossly ineffective in 2024. It is unclear if direct contact with infected birds or mammals, contact with infected feed or water, or contact with fomites in the milking parlor is fueling transmission between and within herds. One California dairy farms had successfully applied technological monitoring of individual cows to identify, quarantine, and treat potential H5N1 infections in their dairy herds for months while neighboring dairies in the Central Valley were reporting H5N1 cases in cows and workers (Mark McAfee, personal communication, December 10, 2024).

Future risk management strategies might include therapeutic and preventative interventions that promote antiviral and immunomodulatory activity, including that provided by the dense and diverse milk microbiota and the extensive array of bioactive components in raw milks, human, and bovine (see Table S7). It is unclear if dairy cows have been treated with the milk glycoprotein lactoferrin that was protective in influenza murine models (Huang et al., 2023). Other murine challenge studies with other influenza A strains documented promising effects of probiotic bacteria: mice administered intranasal H1N1 and oral *Lactobacillus paracasei* (Kim et al., 2023); mice administered *Escherichia coli* Nissle 1917 and a very high dose of H1N1 (Huang et al., 2024); mice administered H3N2 intranasally and *Faecalibacterium duncaniae* intragastrically (Chollet et al., 2024); and mice administered very high doses of H1N1 intranasally and *Lactobacillus mucosae*, *Bifidobacterium breve*, or combination probiotic orally (Lu et al., 2021).

Regarding a related question by a reviewer, multiple sources report that breastfeeding by human mothers infected with influenza A viruses is encouraged, not contraindicated or prohibited by the presence of this and many other viruses (Francese et al., 2023). Well‐documented benefits of raw breastmilk include a diverse array of bioactive components with antiviral activity (see Table S7) that strengthen infant gut and immune systems, and impose a low risk of viral disease transmission for many viruses including influenza A (Blackshaw et al., 2021; Pimentel et al., 2021; Spatz et al., 2021; Francese et al., 2023; Yeo et al., 2022).

The reliance of risk management decisions about samples from bulk milk tanks based solely on PCR testing is problematic, particularly when PCR cycle threshold limits appear subjective or arbitrary, with no reporting of validation data for limits of quantitation for replication competent virus for results from state monitoring of raw milk. At most, PCR testing can provide evidence for presumptive‐positive presence of H5N1 nucleic acid or antigens, not infective virus. The failure of a establishing a causal linkage of the monitoring method to infectivity (and risk) is evident in a recent report of infective influenza virus H1N1 (surrogate for H5N1) in inoculated milk (Zulli et al., 2024). Infective virus persisted for only 2.3 days in inoculated raw milk, significantly shorter than the 57‐day period for which the raw milk was PCR‐positive but noninfective. Two raw milk herds in California's Central Valley were under quarantine solely on the basis of presumptive‐positive PCR testing when infective virus is unlikely to persist or cause influenza via the oral route.

Further consideration of on‐farm interventions for US dairy farms, as well as monitoring for infective virions in cows and milk, appears warranted for herd and worker protection. If the body of evidence cited herein is found inconsistent with the hypothesis that oral transmission of H5N1 to humans is feasible, the need for monitoring of raw milk to protect raw milk consumers may be low.

As per related questions by a reviewer, it is unclear if calves were infected from their mothers, although one inoculation study reported milkborne transmission of H5N1 from inoculated lactating mice to 11 of 24 of their pups (Eisfeld et al., 2024). Typical dairy practice, especially on large dairies, is to separate calves and feed them milk replacer rather than allowing them to suckle.

## 4 CONCLUSIONS

The body of evidence supporting and refuting the hypothesis that avian influenza H5N1 transmits by the oral route to raw milk consumers merits wide deliberation. The extensive body of experimental and observational evidence presented herein and in the Supporting Information is consistent with rare and sporadic transmission of H5N1 by direct contact of dairy workers with infected animals, not by oral transmission. The wisdom of continuing to promote risk perceptions that raw milk is “inherently dangerous” and there is no “downside” to pasteurizing breastmilk (Coleman et al., 2021) and bovine milk (Dietert et al., 2022; Stephenson et al., 2024) also merits deliberation. These risk perceptions are based on factors other than the recent scientific evidence for benefits and risk of raw and pasteurized milks from humans and bovines (Coleman et al., 2021; Dietert et al., 2022; Stephenson et al., 2024).

While concern about avian influenza A mutations and reassortments are warranted, the pandemic potential of H5N1, lacking person‐to‐person and aerosol transmission as main drivers of pandemic potential (EFSA, 2024a), has not increased since the first avian cases were reported in 1996. It is uncertain if future mutations and reassortments might increase severity of the mild infections (eye inflammation, conjunctivitis) reported in dairy workers in 2024. What may be biologically infeasible is that mutation and reassortment could transform H5N1, an enveloped virus with respiratory tropism (Bosch et al., 2018; Lockhart et al., 2022; Rosenke et al., 2024), to a nonenveloped intestinal flu virus with widespread public health burden.

Future incorporation of scientific evidence using robust and transparent risk analysis methodologies may accelerate the development of a coherent knowledge base for more effective communication about and management of H5N1 transmission to consumers and workers. The same lessons for improving risk communication for H5N1 pointed out by risk scholars nearly two decades ago resonate with the 2024 writings of eminent physician‐scientists Drs. Collins and Makary who independently advocated paths to begin countering the current climate of mistrust in the United States. Similarly, acknowledging the “blind spots” that encumber rather than promote human health may be necessary to facilitate a shift to more open, rigorous, and transparent deliberations of the body of scientific evidence to support human health and evidence‐based policies for H5N1.

## DECLARATIONS

MEC is a consultant on medical microbiology and risk analysis who provides expert testimony on the raw milk microbiota benefits and risks, epidemiologic trends, predictive microbiology, and food safety and security. She serves as an unpaid advisor for the Raw Milk Institute and the Canadian Artisan Dairy Alliance.

## Supporting information




**Supporting Table S1**: Evidence on transmission of influenza A H5N1 from inoculation studies with human tissues and cells (in vitro and *ex vivo*). **Supporting Table S2**: Evidence on transmission of influenza A H5N1 from very high dose in vivo inoculation studies with non‐human primates (cynomolgus macaques). **Supporting Table S3**: Evidence on transmission of influenza A H5N1 from inoculation studies with ferrets. **Supporting Table S4**: Evidence on transmission of influenza A H5N1 from very high dose in vivo inoculation studies in cows. **Supporting Table S5**: Evidence on transmission and immune protection (IP) of influenza A H5N1 from in vivo inoculation studies with mice. **Supporting Table S6**: Evidence on transmission of influenza A H5N1 from very high dose in vivo inoculation studies with cats and dogs. **Supporting Table S7**: Some Antiviral Components of Raw Milk and their Multifaceted Health‐Promoting Effects. **Supporting Figure S1**: Weekly Retail Raw Milk Production from 2019 ‐ 2024 from One California Dairy (McAfee 2024)

## Data Availability

The data that support the findings of this study are available on request from the corresponding author. The data are not publicly available due to privacy or ethical restrictions.
