# Supplementary material for: Deliberating the scientific evidence base for influenza transmission to raw milk consumers
Source: Risk Anal. 2025 Jul 15;45(10):2939–55. doi: 10.1111/risa.70077 (PMC12516677; doi:10.1111/risa.70077)
Supplement: Supplementary file 1 — Supporting Table S1: Evidence on transmission of influenza A H5N1 from inoculation studies with human tissues and cells (in vitro and ex vivo). Supporting Table S2: Evidence on transmission of influenza A H5N1 from very high dose in vivo inoculation studies with non‐human primates (cynomolgus macaques). Supporting Table S3: Evidence on transmission of influenza A H5N1 from inoculation studies with ferrets. Supporting Table S4: Evidence on transmission of influenza A H5N1 from very high dose in vivo inoculation studies in cows. Supporting Table S5: Evidence on transmission and immune protection (IP) of influenza A H5N1 from in vivo inoculation studies with mice. Supporting Table S6: Evidence on transmission of influenza A H5N1 from very high dose in vivo inoculation studies with cats and dogs. Supporting Table S7: Some Antiviral Components of Raw Milk and their Multifaceted Health‐Promoting Effects. Supporting Figure S1: Weekly Retail Raw Milk Production from 2019 ‐ 2024 from One California Dairy (McAfee 2024) [file RISA-45-2939-s001.pdf]

Supplementary Materials:

Table S1. Evidence on transmission of influenza A H5N1 from inoculation studies with human tissues and cells (*in vitro* and *ex vivo*)

**Study and Cell Type**	**Administered Dose in Reported Units**	**Evidence of Disease Transmission**	**Reference**
In vitro, human lung cancer cell line Calu-3, (derived from bronchial epithelial cells), 33°C or 37°C	MOI 0.01, 1 hour	No	(Pulit-Penaloza et al. 2024)
In vitro, 1° differentiated nasal epithelium	MOI 0.1 $TCID_{50}$/cell, 3 hours	No	(Richard et al. 2020)
In vitro, 1°differentiated alveolar Type I, Type II cells, macrophages	MOIs 0.01, 2, 1 hour	No	(Yu et al. 2011)
Ex vivo, colorectal tissue, autopsy colonic tissue	10^7 x $TCID_{50}$, 2 hours	No	(Shu et al. 2010)
In vitro, 1° human retinal pigment epithelial cells	MOI 0.01, 2 days	No	(Michaelis et al. 2009)

Multiplicity of Infection = MOI, number of virions inocultated per host cell; $TCID_{50}$ = dose causing infection in 50% of tissue culture cells; 50% tissue culture infectious dose

Table S2. Evidence on transmission of influenza A H5N1 from very high dose *in vivo* inoculation studies with non-human primates (cynomolgus macaques)

Inoculation Route	Administered Doses in Reported Units[a]	Evidence of Disease Transmission	Reference
intrasnasal (mucosal atomization device)<br>intratracheal (instillation via endotracheal tube)<br>orogastric (mouth to stomach, oral cavity and gavage)	10^7 x $TCID_{50}$	Yes, mild[b]<br>Yes, severe[c]<br>No[d]	(Rosenke et al. 2024)
small particle aerosol	5.1 log_{10} pfu	Yes	(Kanekiyo et al. 2023)
fine aerosol at 2 doses	4.9 and 6.72 log_{10} pfu	Yes	(Corry et al. 2022)
combined intrabronchial, oral, intranasal<br>aerosol, with and without bronchoalveolar lavage sampling	6 x 10^6 $TCID_{50}$	Yes<br>Yes	(Mooij et al. 2021)
aerosol; combination intratracheal, intranasal, ocular, oral to tonsils	4 x10^7 pfu	Yes	(Watanabe et al. 2018)
fine aerosol	6.72 log_{10} pfu	Yes	(Wonderlich et al. 2017)
combined intratracheal, intranasal, ocular, oral	$10^{7.8}$ pfu	Yes	(Muramoto et al. 2014)
combined intratracheal, intranasal, conjunctival, tonsillar routes	10^7 pfu	Yes	(Baskin et al. 2009)
combined intrabronchial, oral, intranasal routes	7 x10^6 pfu	Yes	(Cillóniz et al. 2009)
combined intratrachael, tonsillar, conjunctival routes	2.5 x 10^4 $TCID_{50}$	Yes	(Rimmelzwaan et al. 2001)

[a] $TCID_{50}$ = 50% tissue culture infectious dose; pfu = plaque forming units (viral counts). [b] Severely decreased appetite, reluctance to move, open mouth breathing, inflammation, systemic infection, viremia, marked interstitial pneumonia, shedding mainly from oronasal mucosae, histopathological lesions in lungs, serum cytokine increases. [c] Milder signs, significantly lower viremia at day 1, cleared during first week, no evidence of pneumonia, some serum cytokine increases. [d]no signs of clinical illness (some signs attributable to frequent anesthesia), no signs of pneumonia, no viremia, no serum cytokine increases, weaker seroconversion. Note that doses exceed 100,000 viral counts (pfu) or 10,000 times the dose causing infeection for 50% of tissue culture cells.

Table S3. Evidence on transmission of influenza A H5N1 from inoculation studies with ferrets

Study Type (Inoculation or Exposure Route for *In Vivo* Studies)	Administered Dose in Reported Units[a]	Evidence of Disease Transmission	Reference
In vivo, ocular, direct contact at 2 doses	10^3 pfu, 10^6 pfu	Yes	(Belser et al. 2024)
paired with naïve recipient via direct contact	-	Yes	
In vivo (intranasal,	10^6 pfu	Yes	(Eisfeld et al. 2024)
paired with naïve recipient via respiratory droplet)	-	No	
In vivo (intranasal at 4 doses;	$10^{0 \text{ to } 3}$ x $TCID_{50}$	Yes	(Restori et al. 2024)
intranasal,	10^6 x $TCID_{50}$	Yes	
paired with naïve recipient via direct contact,	-	Some[b]	
paired with naïve recipient via respiratory droplet)	-	Not Effective[c]	
In vivo (intranasal; paired with naïve recipient via direct contact,	6 x EID_{50}	Yes	(Pulit-Penaloza et al. 2024)
paired with naïve recipient via respiratory droplet, fomite)	-	No	
In vivo (intranasal,	10^6 pfu	Yes	(Maemura et al. 2023)
paired with naïve recipient via respiratory droplet)	-	No	
In vivo (co-inoculation intranasal and intratracheal donors	10^5 x $TCID_{50}$	Yes,	(Richard et al. 2020)
paired with naïve recipient via aerosol)	-	Yes	
In vitro 1° differentiated nasal epithelial cell cultures	MOI	No	(Zeng et al. 2019)
In vivo (oral feeding at 3 doses in infected meat)	$10^{4.2}$, $10^{6.8}$, $10^{9.2}$ x EID_{50}	Yes	(Bertran and Swayne 2014)
In vivo (ocular, comparisons ocular and intranasal,		Yes,	
paired with naïve recipient via direct contact,	10^6 x EID_{50}	Yes	(Belser et al. 2012)
paired with naïve recipient via aerosol)		No	
In vivo (intranasal,		Yes	
oral (intragastric in liquid medium,	10^6 x EID_{50}	No	(Lipatov et al. 2009)
feeding higher dose via infected meat, intragastric gavage (minced meat))		Yes	

[a] Units: pfu = plaque forming units (viral counts); $TCID_{50}$ = 50% tissue culture infectious dose; EID_{50} = 50% embryonated egg infectious dose. [b] 3 of 4 ferrets infected by direct contact, with delayed signs of infection at days 3, 7, and 9 post-exposure; 3 of 4 with low titers or no seroconversion; [c] Inefficient transmission by airborne route without direct contact, few contacts infected (3 of 12) and slower viral replication kinetics. Note that most doses exceed 1,000 times the dose causing infeection for 50% of tissue culture cells.

Table S4. Evidence on transmission of influenza A H5N1 from very high dose *in vivo* inoculation studies in cows

**Inoculation or Exposure Route**	**Administered Dose in Reported Units[a]**	**Evidence of Disease Transmission**	**Reference**
aerosol to Holstein heifers using mask over nose and mouth,	10^6 x $TCID_{50}$	Yes	(Baker et al. 2024)
intramammary inoculation with massage into udder of lactating cows	10^6 x $TCID_{50}$	Yes	
aerosol to Holstein-Friesian heifers,	$10^{8.5}$ x EID_{50}	Yes	(Kalthoff et al. 2008)
paired with naïve recipients	-	No	

[a] Units: $TCID_{50}$ = 50% tissue culture infectious dose; EID_{50} = 50% embryonated egg infectious dose. Note that doses exceed 1,000,000 times the dose causing infection in 50% of tissue culture cells or infected embryonated eggs.

Table S5. Evidence on transmission and immune protection (IP) of influenza A H5N1 from *in vivo* inoculation studies with mice

Inoculation or Exposure Route	Administered Dose in Reported Units[a]	Evidence of Disease Transmission	Reference
nasopharyngeal, infected milk (1st dose in series),	1.3×10^2 pfu	No	(Eisfeld et al. 2024)
(upper 3 doses of series)	6.5×10^2, 1.3 and 3.25×10^3 pfu	Yes	
intranasal (lower 2 doses in series),	$10^{0,1}$ pfu	Yes (survivors)	
(upper 5 doses in series)	$10^{2,3,4,5,6}$ pfu	Yes	
intranasal	10^3 pfu	Yes	
intranasal to lactating donor and paired with	100 pfu	Yes	
naïve recipient via direct contact	-	No	
nursing pups (24 from 9 liters)	-	Yes, 11 of 24	
nasopharyngeal, infected milk	3×10^6 pfu	Yes	(Guan et al. 2024)
intranasal, +/- dietary lactoferrin	50 µL[b]	Yes, IP	(Huang et al. 2023)
intranasal, 7 doses	$10^{0,1,2,3,4,5,6}$ pfu	Yes	(Maemura et al. 2023)
intranasal	10^3 pfu	Yes	(Ruan et al. 2022)
intranasal or whole body aerosol, dose series	serial 10-fold dilutions[c]	Yes	(Belser et al. 2015)
intranasal<br>intragastric	10^3 EID_{50}	Yes, severe[d]<br>Variable[e]	(Lipatov et al. 2009)

[a] Units: pfu = plaque forming units (viral counts), EID_{50} = 50% embryonated egg infectious dose. [b] Volume, not administered dose specified; virus titer reportedly $10^{8.11}$ [c] Unspecified range of serial 10-fold dilutions. [d] each of three strains caused severe disease with 80-100% mortality. [e] one strain no clinical signs or death, one strain 20% mortality, one strain 100% mortality by intragastric route. Note that most doses exceed 1,000 pfu or 1,000 times the dose causing infection in inoculated embryonated eggs.

Table S6. Evidence on transmission of influenza A H5N1 from very high dose *in vivo* inoculation studies with cats and dogs

Host	Inoculation or Exposure Route	Administered Dose in Reported Units	Evidence of Disease Transmission	Reference
Domestic Cat	intragastric, encapsulated infected chicken liver homogenate	$10^{7.8}$ $TCID_{50}$	Yes	(Reperant et al. 2012)
	oculo-nasalpharyngeal,<br>intravenous,<br>oral encapsulated	10^6 EID_{50}<br>10^6 EID_{50}<br>10^7 EID_{50}	Yes<br>Yes<br>Yes [a]	(Vahlenkamp et al. 2010)
	combined eye, nose, and throat	1, 100, 10,000 EID_{50}<br>10^6 EID_{50}	No<br>Yes	(Vahlenkamp et al. 2008)
	intratracheal donor<br>paired with naïve recipient,<br>oral via infected chicks	2.5×10^4 $TCID_{50}$<br>-<br>$>10^9$ $TCID_{50/g\ tissue}$[b]	Yes<br>Yes<br>Yes[c]	(Rimmelzwaan et al. 2006)
Dog & Cat	oculo-nasalpharyngeal dose to dogs<br>paired with naïve cats and naïve dog via direct contact;<br>oculo-nasalpharyngeal dose to cats<br>paired with naïve dogs via direct contact	10^6 EID_{50}	Yes[d]<br>No[e]<br>Yes, severe[f]<br>No[e]	(Giese et al. 2008)

[a] Evidence gastrointestinal tract portal of entry not replication site; $TCID_{50}$ = 50% tissue culture infectious dose. [b] Chicks infected intratracheally at 2.5×10^4 $TCID_{50}$, euthanized, fed to cats at unreported doses; EID_{50} = 50% embryonated egg infectious dose. [c] H5N1 detected in 3/3 lung, brain, adrenal samples, 1/3 ileum samples, 2/3 duodenum samples, undetected in: tongue, nasal concha, nasal septum, trachea, eyelid, third eyelid, thyroid, salivary gland, tonsil, tracheo-bronchial lymph node, retropharyngeal lymph node, mandibular lymph node, bone marrow, esophagus, stomach, pancreas, jejunum, cecum, colon, and urinary bladder. [d] Mild self-resolving fever, conjunctivitis, PCR-positive pharyngeal swabs (not rectal swabs), plasma and peripheral blood mononuclear cells (PBMC) negative. [e] No clinical signs or positive pharyngeal or rectal swabs, PBMC, or sera. [f] Severe fever, decreased activity, conjunctivitis, labored breathing, 2 of 3 fatal infections, positive pharyngeal and rectal swabs. Note that most doses exceeded 10,000 times the dose causing 50% infection of tissue culture cells or injected embryonated eggs.

Table S7. Some Antiviral Components of Raw Milk and their Multifaceted Health-Promoting Effects

Component	Class	Health-Promoting Effects	Reference
Angiogenin	Protein, enzyme (ribonuclease 5)	Inhibits viral replication, binds actin, endothelial cells, activates proteolytic cascades that degrade basement membranes	(Ng et al. 2015)
Bovine serum albumin	Globular protein	Immunomodulator; opiod agonist; competitive binding to host cell receptors, inhibiting viral infection, complement agonist	(Buey et al. 2023)
Casein	Phophoprotein family	Immunomodulator, anticarciogenic, antioxidant, antimicrobial, antithrombotic, opiod agonist, toxin binding;<br>Antiviral, immomodulator, immunosuppressant	(Buey et al. 2023)<br>(Santos et al. 2024)
Glycomacro-peptide	Derivative of casein	Immunomodulator, antimicrobial, binds viral sialic acid receptors, prevents viral infection and hemagglutination, antiinflammatory, gut microbiome modulator pre-term infants, antiallergic, regulates glucose homeostasis, improves satiety in obese patients;<br>Immunomodulator, antimicrobial, antithrombotic, cytomodulatory, antioxidant, toxin binding	(Santos et al. 2024; Gallo et al. 2024)<br>(Buey et al. 2023)
Lactadherin	Multifunctional phosphorylated glycoprotein	Competetive binding of sialylated and galactosylated glycans with glycoconjugates on host cell, preventing binding of viruses to host cells	(Santos et al. 2024)
Lactoferricin	Derivative of lactoferrin	Immunomodulator, antimicrobial, anticarcinogenic, antioxidant, antithrombitic;<br>Immunomodulator, promotes health of gut microbiota, gut barrier function	(Buey et al. 2023)<br>(Santos et al. 2024)

Component	Class	Health-Promoting Effects	Reference
Lactoferrin	Iron-binding globular glycoprotein in transferrin superfamily	Immunomodulator, antiinflammatory; gut microbiome modulator; Immunomodulator (caspase 3), binds host cells, prevents hemagglutination, infection; Immunomodulator, inhibitor viral infection/replication, prevents cytokine storm; Antiinflammatory, antioxidant, gut microbiota modulator, enhances intestinal barrier function, reduces late-onset sepsis, respiratory/urinary tract infections, antitumor, reduces dysbiosis with chemotherapy, stabilizes gut microbiota, prevents viral infection	(Huang et al. 2023) (Santos et al. 2024) (Zimecki, Actor, and Kruzel 2021) (Gallo et al. 2024)
α-Lactalbumin	Enzyme, globular protein (lactose synthesis)	Antiinflammatory, gut microbiome modulator, benefits growth and development, stabilizes insulin, glucose, lipid metabolism in obese patients, improved intestinal barrier integrity and infant stool consistency Immunomodulator, selective antimicrobial, sleep regulation, mood enhancement, mineral absorption,	(Gallo et al. 2024) (Santos et al. 2024)
α-, β-Lactoglobulin	Proteins	Hypocholesterolaemic; competitive binding to host cell receptors, inhibiting viral infection; opiod-neurotensin agonist, ileum-contracting	(Buey et al. 2023)
Lactoperoxi-dase	Heme-containing glycoprotein, heat-stable enzyme	Antimicrobial (oxidizes thiocyanate or iodine anion generating reactive intermediates), inhibits viral shedding	(Santos et al. 2024)
Lysozyme	Enzyme, lyses bacterial cell walls	Antimicrobial (Gram+/- bacteria, fungi, viruses); increased colonization resistance to pathogens;	(Santos et al. 2024)

Component	Class	Health-Promoting Effects	Reference
		Antiinflammatory, reduces coliforms; modulates, stabilizes gut microbiota; improved recovery diarrhea	(Gallo et al. 2024)
Microbiota (*Lactobacillus Bifidobacteria Pseudomonas*)	Bacteria	Immunomodulator; antagonist; intestinal homeostasis; gut-lung axis; gut-brain axis	(Wang et al. 2023; Santos et al. 2024)
Milk mucin	High molecular weight multifunctional highly glysolated proteins present in fat globule membranes	Inhibits infection by aggregating virus via high sialic acid and sulphate content; prevents viral replication	(Ng et al. 2015)
Oligosaccha-rides	Fucosylated, non-fucosylated neutral, or sialylated compounds with five sugars	Immunomodulator; competes with pathogens for host cell binding sites; reduces allergy, asthma, enteric, inflammatory disease; intestinal homeostasis; promotes gut-brain axis, maintains gut mucosal barrier	(Gallo et al. 2024; Nolan, Rimer, and Good 2020)
Secretory IgA, IgG, IgM	Glycoproteins	Inhibits infectivity by binding viral surface components and preventing replication	(Ng et al. 2015)
Tenacin-C	Large multimeric extracellular matrix glycoprotein	Neutralizes virus by binding viral surface protein, preventing access to host cell, interacts with viral envelope, prevents infection and transmission	(Ng et al. 2015; Santos et al. 2024)

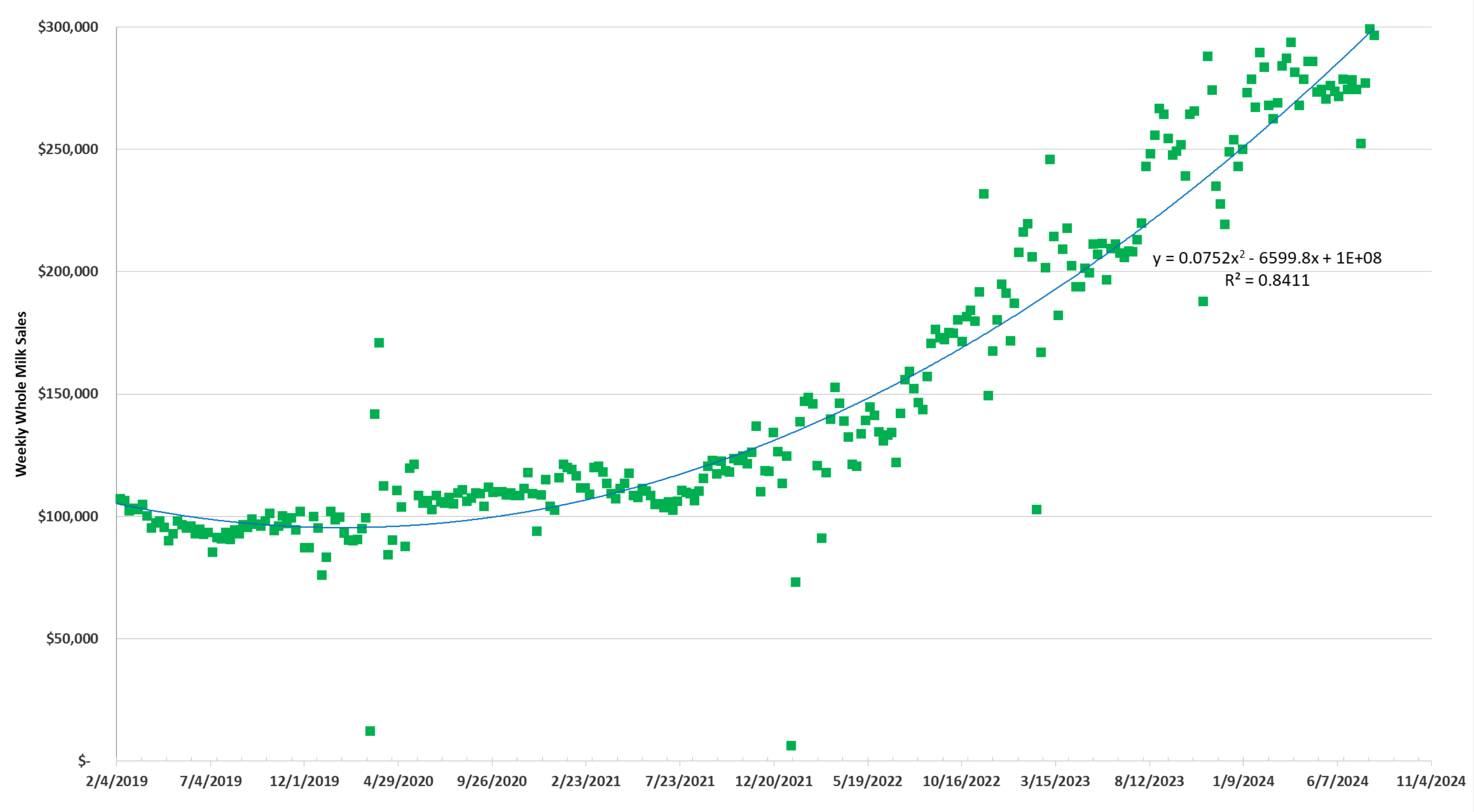

Figure S1. Weekly Retail Raw Milk Production from 2019 - 2024 from One California Dairy (McAfee 2024)

Additional Details from Inoculation Studies

Additional details of experimental evidence from inoculation studies summarized below and in Tables S1-S6. Summaries are provided for experimental treatments and evidence on tissue tropism (T), infectivity (I), replication (R), immune protection (IP) or mortality (M) of influenza A H5N1 (or as specified) inoculated in vivo, ex vivo, or in vitro relevant to informing human health risk analysis.

1.1. Human Studies

The studies conducted with human tissues and cells summarized below (four *in vitro* studies and one *ex vivo* study) provide evidence for infectivity and replication only, not for tissue tropism following natural exposures to H5N1.

1.1.1. Study 1

In primary human nasal epithelium, H5N1 only infected ciliated epithelial cells occasionally and infection did not progress (Richard et al. 2020). In contrast, infection of primary human nasal epithelium by a recombinant virus $H5N1_{AT}$ that carried 9 amino acid substitutions enhancing airborne transmission was higher than H5N1 and more similar to the level of infection and damage to ciliated epithelial cells caused by inoculation with human influenza viruses H1N1 and H3N2.

1.1.2. Study 2

In primary differentiated human alveolar type II and type I cells and macrophages, H5N1 infected and replicated productively with different kinetics (Yu et al. 2011). Also, H5N1 was a potent inducer of proinflammatory cytokines and chemokines, but replication was required since UV or high temperature treatment of H5N1 abolished the induction of proinflammatory activity.

1.1.3. Study 3

In human Calu-3 cells derived from bronchial epithelium of a lung cancer patient, a diverse panel of four H5N1 viruses infected and replicated, though to lower titers at 33°C consistent with upper respiratory tract temperature than at 37°C consistent with lower respiratory tract temperature (Pulit-Penaloza et al. 2024).

In primary human retinal pigment epithelial cells treated with 3 H5N1 isolates, infection and replication to high titers exceeding 10^8 x 50% tissue culture infectious doses (tissue culture ID_{50}) was demonstrated

(Michaelis et al. 2009). H5N1 also induced pro-inflammatory genes. Additional data in this study on human influenza A viruses are not summarized herein.

1.1.4. Study 4

In *ex vivo* colonic tissue mucosal layer cells, H5N1 infected and replicated (Shu et al. 2010). Also, autopsy colon samples from a fatal case with respiratory and GI pathology tested positive for viral NP antigen although Figure 2 D and E did not include error bars or statistical significance results and replication was not demonstrated.

1.1.5. Summary for Human Studies

Overall, *in vitro* and *ex vivo* studies in humans provide no evidence for tropism to target organs, though infectivity and replication in respiratory cells is demonstrated. However, results for replication in *ex vivo* colonic tissue are not definitive, consistent with Mims observation (Mims 1989) that susceptible host cells *in vitro* and *ex vivo* can escape natural infection *in vivo* when anatomical, physiological, chemical, and cellular conditions provide sufficient trophic barriers.

In addition to the data from Table 1 studies, dose-response models are available for *in vivo* human studies for influenza A exposures (intranasal/nasopharyngeal) of attenuated human-adapted influenza viruses H1N1 and H3N2 to human volunteers (Nicas and Jones 2009). Other dose-response models incorporated data for intranasal inoculation of volunteers with H3N3 (5 dose groups), as well as the data from the previous study with H1N1 (4 dose groups) and with H3N2 (9 dose groups) ("QMRAwiki Influenza Dose Response for Intranasal," n.d.). H5N1 virus has not been tested in human volunteers to date.

The available data on transmission pathways for influenza A H5N1 are consistent with the direct physical contact with recently infected dairy and poultry workers (avian and bovine) ("CDC Newsroom" 2024) and perhaps direct contact and inhalation of large droplets or fine aerosols generated by direct contact with infected animals (wild birds and farmed animals including poultry and fur bearing mammals) for historical human cases with avian exposures (Szablewski et al. 2023; Zhao et al. 2023). It is unclear if indirect contact with fomites or aerosols contribute to human disease for dairy or poultry workers.

Some advances in the body of knowledge for influenza A including H5N1 are emerging from experimental studies inoculating virus into *in vivo* animal model systems (nonhuman primates or NHPs, ferrets, and mice) for predicting potential effects in humans. Extrapolations from animal studies may be valid for future human risk analysis models only if key aspects of inoculation, transmission, and replication in animals are representative of processes for natural infections observed in humans.

1.2. Non-human Primate Studies

The studies conducted with non-human primates summarized below (all *in vivo* studies with cynomolgus macaques) provide evidence for tissue tropism, infectivity, and replication for aerosol exposure to the respiratory tract with the exception of the orogastric route (Rosenke et al. 2024).

1.2.1. Study 1

The nature and severity of outcomes in NHPs inoculated at a high dose (10^7 tissue culture ID_{50} for an H5N1 isolate from an infected cow), levels consistent with naturally contaminated milk from infected cows, by three inoculation routes varried markedly (Rosenke et al. 2024). Intranasal inoculation mimicing upper respiratory transmission caused mild systemic illness, with localized replication and clearance. Intratracheal inoculation of the same dose and strain mimicing lower respiratory transmission caused severe and fatal systemic illness. Subclinical outcomes, with limited infection, replication, and seroconversion, were reported for orogastric inoculations ($1.7x10^6$ tissue culture ID_{50} orally and $8.3x10^6$ tissue culture ID_{50} by the intragastric gavage). Highest titers of viral RNA were detected in blood of the intratracheal group at euthanasia. Lower titers detected in the intranasal group were cleared in the first week. No viral RNA was detected in the orogastric group.

1.2.2. Study 2

Inoculation at ~5.1 log_{10} pfu by the aerosol route induced a strong febrile response and acute respiratory disease, with 4 of 6 infections fatal (Kanekiyo et al. 2023)

1.2.3. Study 3

Severe respiratory outcomes and inflammatory cascade in the lungs of 6 animals were reported at a lethal dose (6.72 log_{10} pfu), and mild respiratory illness at a lower dose (4.9 log_{10} pfu) in 3 animals (Corry et al. 2022). Depletion of alveolar macrophages and neutrophils in bronchoalveolar lavage (BAL) correlated with virus load in lethal and mild dose-groups.

1.2.4. Study 4

Inoculation at $6x10^6$ tissue culture ID_{50} by three different routes (combined intrabronchial, oral, intranasal; aerosol; or aerosol with bronchoalveolar lavage at days 2, 4, and 7 post-inoculation) (Mooij et al. 2021)

1.2.5. Study 5

Both aerosol exposure and 'conventional' exposure (intratracheal, intranasal, ocular, and oral) at the same total dose ($4x10^7$ pfu/mL) initiated productive infection in groups of 4 animals as measured by comparable titers in nasal swabs days 1-3 and similar lung pathology at day 4 (nasal mucosa, trachea, bronchus, tonsils, mediastinal lymph nodes). No histopathological changes or detection of viral antigen was observed in tissues other than trachea (mild inflammatory cell infiltration, no replication) and lung (pulmonary edema, inflammatory cell infiltration, desquamation of alveolar epithelial cells, and viral antigen in bronchial and alveolar epithelium) (Watanabe et al. 2018).

1.2.6. Study 6

Severe respiratory outcomes, fulminant pneumonia, inflammatory cascade in the lungs of 7 animals, and acute respiratory distress syndrome (ARDS) were reported at a lethal aerosol dose (6.72 log_{10} pfu) in a head-only chamber, with intensely hemorrhagic lungs, intense replication in cells lining most alveoli (alveolar epithelial cells and alveolar macrophages), and loss of epithelial barrier function (Wonderlich et al. 2017). High titers were observed in lungs at necropsy, intermittent low titer recovery in nasal washes, and consistent recovery in BAL.

1.2.7. Summary for Nonhuman Primate Studies

Overall, non-human primate studies included few animals per treatment group and predominantly very high lethal dose treatments that offer limited utility for extrapolating to natural human exposures. However, the observations of lack of clinical disease from orogastric inoculation (Rosenke et al. 2024), both mild and severe illness outcomes and demonstration of the dose-dependency of disease severity (Corry et al. 2022), as well as mechanisms consistent with human pathogenesis, are relevant for potential extrapolation to humans. The lethal dose studies (Wonderlich et al. 2017; Watanabe et al. 2018) are also relevant to severe human disease, particularly as these studies demonstrate a common mechanism driving ARDS and fatal effects in NHPs and humans: high viral doses inducing profound interferon responses that promoted innate immune system cascades in the lung, amplified inflammatory processes,

and caused immunopathology, with severe and fatal effects in the respiratory tract (Corry et al. 2022). Authors of this study noted that host response to high viral doses (cytokine storm causing immunopathology), not replication of the virus, appears to drive severe outcomes. The multiple dose-study in non-human primates (Corry et al. 2022) is consistent with principles of dose-response assessment and recent human surveillance studies documenting asymptomatic occupationally exposed workers (Oliver et al. 2022). Authors of another study (Wonderlich et al. 2017) note that fatal disease appears driven by direct viral effects in the lung alveoli.

The 10 *in vivo* NHP studies (cynomolgus macaques) summarized in Table 2 included few animals per treatment group and thus should be interpreted with caution. Overall, these studies all provide evidence for respiratory transmission at high doses via aerosols or instillations including intranasal, intratracheal, and combinations of different potential routes (e.g., intratracheal, intranasal, ocular, oral). The NHP studies reveal mechanisms consistent with human pathogenesis and outcome. The body of evidence from NHPs provides strong evidence of transmission relevant for potential extrapolation to humans, particularly: i) observations of lack of clinical disease from orogastric inoculation (Rosenke et al. 2024); ii) lack of gasterointestinal infection from combined inoculation routes in six studies (Rimmelzwaan et al. 2001; Baskin et al. 2009; Cillóniz et al. 2009; Muramoto et al. 2014; Mooij et al. 2021; Watanabe et al. 2018); and both mild and severe illness outcomes and demonstration of the dose-dependency of disease severity (Corry et al. 2022).

The lethal dose studies (Wonderlich et al. 2017; Watanabe et al. 2018) are also relevant to severe human disease, particularly as these studies demonstrate a common mechanism driving acute respiratory distress syndrome and fatal effects in NHPs and humans: high viral doses inducing profound interferon responses that promoted innate immune system cascades in the lung, amplified inflammatory processes, and caused immunopathology, with severe and fatal effects in the respiratory tract (Corry et al. 2022). Authors of this study noted that host response to high viral doses (cytokine storm causing immunopathology), not replication of the virus, appears to drive severe outcomes. The multiple dose-study in NHPs (Corry et al. 2022) is consistent with principles of dose-response assessment and recent human surveillance studies documenting asymptomatic occupationally exposed workers (Oliver et al. 2022). Authors of another study (Wonderlich et al. 2017) note that fatal disease appears driven by direct viral effects in the lung alveoli.

In contrast, the most recent study reported asymptomatic infection by oral inoculation, including: i) lack of clinical disease with some evidence of localized infection and viral clearance; ii) no histopathological lesions, systemic disease, or pneumonia; and iii) nondetectable virus in blood and rectal swabs for oral exposure from mouth to stomach termed orogastric (Rosenke et al. 2024). Outcomes reported in the same study from inoculation at the same dose for the intranasal route were mild to moderate self-limiting disease with clearance, and for the intratracheal route, severe fatal pneumonia (Rosenke et al. 2024). These authors suggested that consumption of contaminated food or liquid is likely self-limiting, compared to upper and lower respiratory system disease after droplet and aerosol exposures.

1.3. Ferret Studies

The 10 studies conducted with ferrets summarized in Table 3 (9 *in vivo* studies by aerosol, intranasal, intratracheal, oral, or ocular routes, plus one *in vitro* study) provide strong evidence for transmission via direct contact with infected animals. Overall, ferret studies included few animals per treatment group and predominantly very high lethal dose treatments that offer limited utility for extrapolating to natural human exposures.

1.3.1: Study 1

Inoculation of human H5N1 isolate (Chile/25945) at high dose (10^6 pfu) and lower dose (10^3 pfu) by the ocular route induced productive systemic and fatal infection, as well as via direct transmission to co-housed naïve recipients (Belser et al. 2024). One of 6 inoculated ferrets survived lower dose challenge, and two of 6 contact animals survived.

1.3.2: Study 2

Respiratory tropism with highest titers in nasal turbinates was documented in 4 ferrets inoculated intranasally with 10^6 pfu of H5N1 strains (cow or VN1203) or H1N1 (Eisfeld et al. 2024). High titers were also observed for H5N1 strains in trachea and lung, higher and more consistent titers in liver, brain, colon, and mammary gland for VN1203. Notably, the cow strain was detected at moderate titers in brain, spleen and colon, but was not detected or present at low titers in teat and mammary glands. Authors note that tropism to mammary tissue is not unique to the recent bovine isolate. For ferrets inoculated as above and paired with naïve recipients in adjacent cages without direct contact, H1N1 but not H5N1 transmitted efficient via respiratory droplets in ferrets.

1.3.3: Study 3

Intranasal inoculation at 3 or 4 doses (10^0 to 10^3) for 4 ferrets per tissue culture ID_{50} dose (doses in pfu not reported) generated an estimated ID_{50} of 3 tissue culture ID_{50} for H5N1 (isolate from mink), 5 tissue culture ID_{50} for H3N3, and 1 tissue culture ID_{50} for H1N1 (Restori et al. 2024). Animals at the three higher doses all became infected, lost weight, and exhibited severe clinical disease. For assessing transmission from infected ferrets, 4 donors were inoculated intranasally with 10^6 tissue culture ID_{50} and paired with naïve recipient animals, either co-housed (direct contact test) or housed in adjacent cages without direct contact (respiratory test). Some evidence suggests that H5N1 may effectively transmit via direct contact (3/4 infected, but 2 of these 3 had low titers or no seroconversion). H5N1 does not efficiently transmit via airborne route (1/4 and 2/4 animals infected via airborne exposure in the absence of direct contact).

1.3.4: Study 4

Severe respiratory outcomes and inflammatory cascade in the lungs of 9 ferrets via intratracheal inoculation at a lethal dose (6 times embryonated chicken egg ID_{50}, dose in pfu not reported) (Pulit-Penaloza et al. 2024). Infection and replication were demonstrated in respiratory tissues, with high titers in nasal turbinates, trachea, and lungs and extrapulmonary spread. Inoculated animals paired with naïve ferrets transmitted infection only via close direct contact, not by respiratory droplets or fomites in the absence of close direct contact. Authors concluded that the viral load in air from a lethally infected ferret was insufficient to transmit to ferrets housed in adjacent cages, despite detection of viral RNA in air. Similarly, although viral RNA was detected on cage walls from infected ferrets in the fomite transmission model, no transmission was observed to naïve ferrets then housed in contaminated cages.

1.3.5: Study 5

Ferrets (3-8 per group) inoculated intranasally with 10^6 pfu of 2 H5N1 strains and an H1N1 strain developed highest titers in respiratory tissues (Maemura et al. 2023). H5N1 distributed systemically to liver, colon, heart, brain, kidney, spleen, and feces. No H5N1 transmission or seroconversion was documented to naïve recipient animals in adjacent cages restricting direct contact, but transmission of H1N1 to recipient ferrets was documented.

1.3.6: Study 6

Transmission from donor ferrets (co-inoculated intranasally and intratracheally) with high H5N1 doses (10^5 tissue culture ID_{50}; dose in pfu not reported) of tagged and untagged virus to recipient ferrets (in

adjacent cages without direct contact) was generated from upper respiratory tract (nasal epithelium) of the infected donors (Richard et al. 2020). Transmission was defined as detection of two consecutive nasal or throat swabs in recipient ferrets with a threshold value real time PCR (Ct value) <35. H5N1 appeared to infect and replicate in nasal respiratory epithelium rather than the nasal turbinates. Comparisons were made with recombinant virus $H5N1_{AT}$ that carried 9 amino acid substitutions enhancing airborne transmission. Authors concluded that H5N1 replication in the upper respiratory tract (nasal respiratory epithelium) is a driver of airborne transmission in ferrets.

1.3.7: Study 7

Infection and replication of inoculated H5N1 occurred primarily in non-ciliated cells in primary ferret nasal epithelium, with replication higher at 37°C typical of the lower respiratory tract than at 33°C typical of the upper respiratory tract (Zeng et al. 2019). H5N1 induced higher expression of immune mediator genes and caused greater damage than observed in primary ferret nasal epithelium inoculated with human adapted H1N1. Distribution of sialic acid receptors in ferret cells was characterized along with replication and immune responses to H5N1 and H1N1 at relevant physiological temperatures. Authors conclude that H5N1, unlike H1N1, is not adapted to replicate efficiently at lower temperatures of nasal passages, likely limiting transmission of H5N1 among mammalian species.

1.3.8: Study 8

Morbidity, mortality, seroconversion, $ID_{50,}$ and LD_{50} were assessed for groups of 3 ferrets dosed by oral feeding of infected meat at low, medium or high doses (~$10^{4.2}$ egg ID_{50}, ~$10^{6.8}$ egg ID_{50}, or ~$10^{9.2}$ egg ID_{50}, respectively; dose in pfu not reported) of two clades (Mong/05 and VN/04) (Bertran and Swayne 2014). Mong/05 was both less infective and less virulent than VN/04 (respectively, ID_{50} 4.9 and 7.5 log10 egg ID_{50}/0.1 mL; respectively, LD_{50} >9.2 and 8.9). For both clades, no morbidity or mortality was observed at the low dose, as well as at the medium dose for clade VN/04. Mong/05 at the medium and high doses caused morbidity in 1/3 ferrets as well as seroconversion in 6/6 ferrets. Seroconversion was reported for clade VN/04 in 1/3 and 1/1 ferrets at medium and high dose, respectively. No fatalities were observed for 9 animals dosed with clade Mong/05, while 2/3 fatalities with severe respiratory and systemic lesions were observed at high dose for clade VN/04. Two additional clades of H5N1 fed to 2 ferrets each at high doses in infected meat (~$10^{9 \text{ to } 9.2}$ egg ID_{50}) seroconverted and developed respiratory lesions and pneumonia, as did H7N3 but not H7N7. Authors note that much higher H5N1 doses are needed to infect by the oral route versus intranasal route.

1.3.9: Study 9

Transmission to the upper and lower respiratory tract via the intranasal route for 4 different clades of H5N1 was demonstrated at high dose (10^6 x egg ID_{50}; dose in pfu not reported) with some differences in pathogenicity by clade, while the same dose via the intragastric route in liquid was nonpathogenic for all 4 clades (Lipatov et al. 2009). Oral exposure by feeding a higher dose (~$10^{9.5}$ egg ID_{50}) of three clades in meat caused mild upper respiratory infection or systemic infection. Intragastric inoculation of one clade at high dose (~$10^{8.3}$ egg ID_{50}) in minced meat was nonpathogenic to one ferret and lethal to 3 others exhibiting systemic disease. Authors note that the dose of H5N1 needed to infect ferrets orally was much higher than by respiratory exposure.

1.3.10: Study 10

Transmission of 2 H5N1 strains by ocular inoculation with 10^6 egg ID_{50} (dose in pfu not reported) was demonstrated in ferrets, two of 3 surviving for one strain and all 3 dying for the other (Belser et al. 2012). Upper respiratory tract infection that progressed to lower respiratory and systemic infection was noted, similar to intranasal transmission but with delayed kinetics of dissemination and neural damage. Moderate titers of H5N1 were detected in nasal washes for 5 of 6 ferrets, and low titers were reported in corneal washes for 1 of 3 ferrets for each H5N1 strain. For one H5N1 strain, corneal washes from 3 ferrets were negative, and for the other strain, 2 of 3 samples had low titer. Ferret corneal epithelial sheets predominantly expressed α2-3 sialic acids, with weaker expression of α2-6 sialic acids at the corneal surface, similar to human corneal and conjunctival tissue. Further testing for two H5N1 strains (also H1N1 and H3N2, not H5N1) revealed efficient replication in the upper respiratory tract following intraocular inoculation and lead to efficient transmission by direct contact but not by respiratory droplets.

1.3.11: Summary for Ferret Studies

Overall, ferret studies included few animals per treatment group and predominantly very high lethal dose treatments that offer limited utility for extrapolating to natural human exposures. However, particularly relevant results from ferret studies for potential extrapolation to humans include the following: i) H5N1, unlike H1N1, is not adapted to replicate efficiently at lower temperatures of human nasal passages, likely limiting transmission of H5N1 among humans (Zeng et al. 2019); ii) 10^6 egg ID_{50} intragastric inoculation (dose in pfu not reported) was nonpathogenic, while the same dose caused fatalities by the intranasal route (Lipatov et al. 2009); iii) oral doses orders of magnitude higher than

intranasal doses were required to transmit influenza to ferrets, but via respiratory and systemic disease (Bertran, Dolz, and Majó 2014; Lipatov et al. 2009), not GI disease typical of intestinal flu (Lockhart, Mucida, and Parsa 2022); iv) viral loads in air and on cage walls, though detectable from contact with intranasally inoculated animals, were insufficient to transmit to naïve donor ferrets (Pulit-Penaloza et al. 2024; Restori et al. 2024); and v) 10^6 egg ID_{50} ocular doses (dose in pfu not reported) caused fatal respiratory and systemic infections at delayed kinetics observed for intranasal dosing (Belser et al. 2012). Further, we note that even the lowest dose treatment from the multi-dose feeding study (Bertran, Dolz, and Majó 2014) could be considered a moderate dose, as it was orders of magnitude above the limit of detection and the dose causing egg ID_{50} for the H5N1 strain. Taken together, these data suggest limited transmission and replication in the human upper respiratory tract without direct contact of infected animals.

However, particularly relevant results from ferret studies for potential extrapolation to humans include the following: i) H5N1, unlike H1N1, is not adapted to replicate efficiently at lower temperatures of human nasal passages, likely limiting transmission of H5N1 among humans (Zeng et al. 2019); ii) a high dose via intragastric inoculation (dose in viral counts or plaque forming units (pfu) not reported) was nonpathogenic, while the same dose caused fatalities by the intranasal route (Lipatov et al. 2009); iii) oral doses orders of magnitude higher than intranasal doses were required to transmit influenza to ferrets, but causing respiratory and systemic disease (Bertran, Dolz, and Majó 2014; Lipatov et al. 2009), not GI disease typical of intestinal flu (Lockhart, Mucida, and Parsa 2022); iv) viral loads in air and on cage walls, though detectable from contact with intranasally inoculated animals, were insufficient to transmit to naïve donor ferrets (Pulit-Penaloza et al. 2024; Restori et al. 2024); and v) high ocular doses (dose in pfu not reported) caused fatal respiratory and systemic infections that took longer to develop relative to intranasal administration (Belser et al. 2012).

Further, note that even the lowest dose treatment from the multi-dose feeding study (Bertran, Dolz, and Majó 2014) could be considered a moderate dose, as it was orders of magnitude above the limit of detection and the dose causing half of the treated animals to get infected for the H5N1 strain. Taken together, these data suggest limited transmission and replication in the human upper respiratory tract without direct contact of infected animals.

1.4. Cow Studies

One *in vivo* study was conducted in Holstein cows and another in Holstein-Friesian cows (Table 4).

Two *in vivo* studies with Holstein-Friesians (Kalthoff et al. 2008) and Holsteins (monitored with ear-tag accelerometer sensors and thermal microchips) (Baker 2024) documented effects of inoculation of H5N1 isolates from cat (A/cat/Germany/R606/2006) and dairy cow (A/dairy cattle/Texas/2486 008749-002/2024: TX/24: NCBI PP755581- PP755588; B3.13 strain), respectively. Neither study demonstrated presence of viral antigen or live virus in fecal swab samples, jejunum, or feces of inoculated animals.

1.7. 1. Study 1

One study (Baker 2024) provided evidence for tissue tropism, infectivity and replication for a bovine strain of H5N1 inoculated by the aerosol route in 4 heifer calves that showed no clinical signs, as well as evidence of infectivity and replication for the intramammary route in 2 lactating cows with signs of mastitis and milk thickening and color changes from inoculated quarters at 2 to 14 days post inoculation. Lung tissues from 2 of 4 heifers were positive for viral antigen. Other heifer tissues were non-detectable for viral antigen.

The 2 lactating cows inoculated by the intramammary route also showed mild clinical signs (decreased rumen motility and milk production, lethargy, reduced feed intake, self-resolving watery diarrhea or dry feces, and intermittant nasal discharge, with signs of recovery from 14-24 days. However, All heifers and lactating cows seroconverted during the study.

Viral antigen was not detected in rectal swabs, jejunum, or feces of inoculated animals. No fecal or blood swabs were positive during the study. One sample from nasal, oropharyngeal, and ocular swabs was positive for viral antigen on day 3, and all subsequent swabs were negative. Viral nucleoprotein antigen was detected in: respiratory epithelial cells lining the conducting airway by IHC for inoculated heifers; and cytoplasm and nucleaus of epithelial cells lining secretory alveoli in inoculated mammary quarters. The presence of replicating virus was demonstrated by immunohistochemistry in respiratory epithelial lining bronchioles of heifers and in milk from inoculated quarters. H5N1 was shed at low Ct values (high doses) in milk, and evidence of replication was demonstrated by the amount and duration of virus shedding in milk. Periodic inoculation of milk samples into the egg assay demonstrated live virus in milk from inoculated quarters of lactating cows prior to day 10.

Viable virus was reported detected in milk samples up to 10 days post inoculation by reverse transcription real time quantitative PCR for viral RNA (H5N1 matrix gene), although only Ct results (15 to 27 cycles), not

estimation of plaque-forming units, were reported in Figure 2D. Live virus was not isolated from milk by egg inocultation studies from days 12-24.

The Holstein study provides evidence for disease transmission, as well as tissue tropism, infectivity and replication for a bovine strain of H5N1 (A/dairy cattle/Texas/2486 008749-002/2024: TX/24: NCBI PP755581- PP755588; B3.13 strain) inoculated by the aerosol route (heifer calves), and infectivity and replication for the intramammary route (lactating cows) (Baker et al. 2024). No overt signs of illness were reported for four heifers except transient increased nasal secretions.

Inoculated lactating cows developed mild illness including these clinical signs: changes in milk color and consistency consistent with mastitis in inoculated quarters only; declines in rumen motility and milk production; lethargy; reduced feed intake; self-resolving watery diarrhea or dry feces; and intermittant clear nasal discharge. At necropsy, 47 tissues and samples were analyzed for viral antigen for the inoculated heifers, and 51 tissues and samples (including an additional four mammary tissue samples) were analyzed for inoculated lactating cows.

Viral antigen was not detected in rectal swabs, jejunum, or feces of inoculated animals. Inoculated cows showed mild clinical signs for 7-14 days and appeared to be recovering from 14-24 days. Viable virus was detected in milk samples up to 10 days post inoculation. All heifers and lactating cows seroconverted during the study.

Bovine inoculations in the Holstein study generated signs of illness similar to the mild self-resolving illness observed in naturally infected herds (Baker et al. 2024). Aerosol inoculation through a nose and mouth mask confirmed tissue tropism to bronchial epithelial cells, and infection and replication predominantly in the upper respiratory system. Though it seems likely that some of the inoculum delivered through the mask may have been swallowed, no evidence of infection and replication was observed in GI tract. Neither was virus detected in urine and feces.

For the lactating cows injected via canula into the mammary gland (Baker et al. 2024), H5N1 infection did not progress beyond the mammary glands and supramammary lymph node based on negative results (polymerase chain reaction thresholds (Ct) greater than 35) from testing of 48 of 51 tissues and samples listed above.

The Holstein-Friesian cows in the second study included in Table 4 were inoculated at high aerosolized dose of an H5N1 isolate from a cat (Kalthoff et al. 2008). Only nasal swabs and serological samples, not tissue samples, were analyzed using methodogy and reagents that are somewhat outdated. Thus, the study provides evidence for aerosol transmission, but no evidence on tissue tropism, infectivity and replication.

Five heifers inoculated with 2 x 10^6 times tissue culture ID_{50} (dose in pfu not reported) by aerosol delivery through a mask covering nostrils and mouth displayed no overt signs of illness except transient nasal secretions. Virus was isolated from nasal swabs in one heifer, and from an oropharyngeal swab in another heifer. Virus was not isolated from nasal, orypharyngeal, ocular swabs or saliva samples for at least three of four heifers. At necropsy (7 days post inoculation), only lung tissue was positive viral antigen for one heifer, and all tissues were nondetectable (cycle threshold >35) at both 7- and 20-day necropsy. Minimal multifocal pulmonary consolidation was reported for one of four heifers. The following tissues and samples from authors' Supplemental Table 2 were analysed for inoculated heifers (except mammary) and inoculated lactating cows:

abomasum, blood, brainstem, brisket, bronchoalveolar lavage fluid, cerebellum, cerebrum, conjunctiva, descending colon, diaphragm, fecal swab, feces, heart, ileum, jejunum, kidney, liver, lymph nodes (ileocecal, inguinal mandibular, mesenteric, parotid, popliteal, retropharyngeal, supramammary, tracheobronchial), lung (accessory lobe, caudal, cranial lobe, middle lobe), mammary, ocular fluid (aqueous, vitreous), omasum, pancreas, reticulum, rumen, rumen content, rump (gluteus medius; minimus, biceps femoris), spiral colon, spleen, tenderloin, thymus, trachea, tracheal swab, turbinate, urine). Some evidence of replication was observed in the bronchial respiratory epithelium (type II pneumocytes and alveolar macrophages.

No fecal or blood samples were positive at any point during the study.

Two cows inoculated at 10^5 times tissue culture ID_{50} (dose in pfu not reported) within each of two quarters using a teat canula into the mammary glad (with massage into the teat sinus) developed signs including:

positive for California mastitis test with changes in milk color and consistency consistent with mastitis in inoculated quarters only; declines in rumen motility and milk production; lethargy; reduced feed intake; self-resolving watery diarrhea or dry feces; intermittant clear nasal discharge.

Neurological signs were not observed. Viable virus was not detected in pooled milking machine bucket or individual quarter milk samples by 12 days post-inoculation. At necroscopy (24 days post inoculation), viral antigen was detected only in mammary tissues from inoculated quarters and supramammary lymph nodes. Remaining macroscopic evaluations (thoracic cavity, abdominal cavity and cranium) and immunohistology for H5N1 nucleoprotein antigen (nasal turbinate, trachea, lung, tracheobronchial lymph node, samples listed above) were negative or unremarkable. Evidence of gross pathology and histologic lesions was limited and inconsistent between animals. No fecal or blood samples were positive. All inoculated cows seroconverted by immunohistochemistry targeting antibody against viral antigen.

Bovine inoculations in this study generated signs of illness similar to those observed in naturally infected herds (Burrough et al. 2024). Aerosol inoculation through a nose and mouth mask confirmed tissue tropism to bronchial epithelial cells, and infection and replication predominantly in the upper respiratory system. Though it seems likely that some of the inoculum delivered through the mask may have been swallowed, no evidence of infection and replication was observed in GI tract or urine and feces. These finding are from small numbers of inoculated animals, and extrapolation should not be undertaken without additional evidence. However, from observational studies documenting mild disease (and likely limited replication) for dairy cows, a testable hypothesis for future research is that cow manure is an unlikely vehicle to spread H5N1 in the environment.

For the lactating cows injected via canula into the mammary gland, infection did not progress beyond the mammary glands and supramammary lymph node based on negative results (polymerase chain reaction threshold results (Ct) greater than 35) from testing of 51 tissues and samples listed above. Another testable hypothesis for future research is that virus transmits between cows and herd via milk or milking machines.

1.7. 2. Study 2

A H5N1 isolate from a cat was administered intranasally to four Holstein-Frisian heifers at a high dose of aerosolized virus ($10^{8.5}$ times egg ID_{50}; dose in pfu not reported) (Kalthoff et al. 2008). Inoculated heifers were housed with two naïve (uninoculated) heifers throughout the 91-day observation period. Authors reported no clinical signs in inoculated and contact heifers. Nasal swab and serology samples, but no tissue samples, were analyzed in this study. Although some asymptomatic heifers reportedly shed infective virus in nasal swabs on day 1 and 2, the PCR cycle threshold ranges reported in Table 1 were

not appear to be in quanitifiable range (<35) for two heifers on day 1 and all 4 heifers on day 2. Serological tests for hemaglutinin and nucleoprotein inhibition were negative for contact heifers throughout the experiment and for inoculated heifers at 7 days postinoculation, with inconsistent results for days 14, 21, 28, and 91. Only at day 28 were samples positive by all three serological tests (inhibition of nucleoprotein and hemmaglutin, and virus neutralization). Some seroconversion was documented in 5 of 6 heifers 3 months after inoculation.

1.4. Mouse Studies

The 7 recent *in vivo* studies conducted with mice summarized below provide evidence for tissue tropism, infectivity, and replication for multiple inoculation routes (intranasal, intragastric, or nasopharyngeal (back of the throat and nasal cavity)).

Key findings include the following.

1.4.1: Study 1

Ten anesthetized BALB/cJ mice treated by nasopharyngeal inoculation (back of the throat, and nasal cavity documented in previous study) with 4 doses of naturally contaminated milk (A/dairy cattle/Kansas/SM-3/2024; 1.3×10^2 pfu, 6.5×10^2 pfu, 1.3×10^3 pfu, and 1.3×10^3 pfu) did not seroconvert (Eisfeld et al. 2024). Replication was observed at the two higher doses in nasal turbinates, lung and brain (intestine not analyzed). Replication in the lower two nasopharyngeal doses were sporadic or not observed, and an ID_{50} for this route was not estimated by the authors.

Intranasal inoculation to groups of 5 animals at 7 doses of cow-H5N1 (10^0 to 10^6 pfu) resulted in an estimated LD_{50} = 31.6 pfu for the intranasal route. All 5 mice in the lowest dose group and some animals in 10^1 and 10^2 pfu dose groups survived, and 100% lethality was observed for doses $\geq 10^3$ pfu.

Intranasal inoculation of 10 mice per group at 10^3 pfu of 2 H5N1 strains yielded similar tissue tropism with highest titers in lung, trachea, nasal turbinates, brain, and mammary gland, and detection at moderate titers in intestine for both the cow and human H5N1 isolates. Authors note that tropism to mammary tissue is not unique to the recent bovine isolate. For the H1N1 strain, replication was limited to respiratory tissues.

Intranasal inoculation with 100 pfu to 6 lactating mice demonstrated replication in lung, nasal turbinates, and brain in all inoculated mice, though virus was not detected in milk of 3 or 4 mice. H5N1 transmission was demonstrated in 1 to 5 pups per litter for 4 of 6 mice. No transmission was demonstrated for remaining two litters of pups or to naïve adults co-housed with inoculated adults.

1.4.2: Study 2

Five anesthetized BALB/cJ mice treated by nasopharyngeal inoculation (back of the throat and nasal cavity) with a high dose (3 x 10^6 pfu) of H5N1 in naturally contaminated raw milk (sample #93) developed ruffled fur and lethargy one day post inoculation, progressing to respiratory and systemic infection; no infection or replication of intestinal tissues or fecal swabs was demonstrated (Guan et al. 2024). Highest titers were reported in lung, trachea, nasal turbinates, and spleen, with nondetectable titers in eye, teat, feces, and intestine (Figure S3). Mammary glands in 2 of 5 mice were viral positive, but replication in mammary glands was not demonstrated.

One study listed only three of 11 raw milk samples (#90, #93, #115) in Table S1 that yielded quantitative viral titers (Guan et al. 2024), but the Ct value estimated for the hemagglutinin gene for one of the 3 samples (#115) was unacceptable (Ct > 35), as noted elsewhere in the literature (Baker 2024). Six of 11 raw milk samples in addition to the two samples with valid quantitative titers amplified sufficiently in egg or canine kidney (Madin-Darby canine kidney or MDCK) cells to confirm presence of very low levels of live virus in naturally infected raw milk (limit of detection=10 pfu/mL). Three raw milk samples with acceptable Ct values for viral genes (#102, #111, #116) did not permit amplification in either eggs or MDCK cells.

1.4.3: Study 3

Five BALB/c mice intranasally infected with H5N1 (dose not reported) and fed standard diet supplemented with the antimicrobial peptide lactoferrin (10 mg/day) regained weight loss after initial infection and survived, while the group inoculated and fed standard diet suffered progressively severe weight loss, hair loss and dull hair color, diarrhea, lethargy, tachypnea, and anorexia (Huang et al. 2023). Lactoferrin reduced influenza-induced histopathology in mouse lung and colon tissues or enhanced repair via modulation of intestinal and lung immune systems through complex and extensive synergistic interactions with the mucosal immune system, the gut microbiota, inflammatory factors, and the intestinal barrier.

1.4.4: Study 4

Four BALB/c mice intranasally infected with 3 H5N1 strains at 7 doses (10^0 to 10^6 pfu) resulted in an estimated LD_{50} of 48, 30, and 2.2 pfu for the intranasal route (Maemura et al. 2023). Tissue tropism after intranasal inoculation of groups of 10 mice with 3 H5N1 strains at 10^3 pfu predominantly affected respiratory tissues at highest titers, with some systemic spread in heart, brain, and spleen, and more sporadic distribution with non-detectable or low titers for liver, colon, kidney and heart.

1.4.5: Study 5

Anesthetized C57BL/6 mice (4 to 10 per group) inoculated intranasally with 1,000 pfu developed widespread edema and inflammatory cell infiltration in lungs, associated with decreased intercellular junction proteins in alveolar and bronchial epithelial cells and loss of alveolar epithelial barrier function (Ruan et al. 2022). Additional mechanistic research using multiple *in vitro* cell systems supported interpretation of the *in vivo* experiments with mice.

1.4.6: Study 6

Infective and lethal doses were estimated for BALB/c mice anesthetized and inoculated intranasally or exposed to aerosols in a whole body chamber at 10-fold serial dilutions of H5N1, H7N9, and H1N1 strains (Belser et al. 2015). The ID_{50} for H5N1 was 8.9 pfu for aerosol and 15.8 pfu for intranasal routes. The LD_{50} was 15.8 pfu for aerosol and 88.9 pfu for intranasal routes. H5N1 appeared less infective (higher ID_{50}) than H1N1 and more virulent (lower LD_{50}) than both H1N1 and H7N9 in this study.

1.4.7: *Study 7*

Groups of 4 BALB/c mice inoculated via both the intranasal and intragastric routes with 4 Asian H5N1 clades at 10^3 egg ID_{50} (dose in pfu not reported), developed marked differences in transmission and pathogenicity (Lipatov et al. 2009): one clade was nonpathogenic via both routes; one clade was pathogenic only by the intranasal, not intragastric, route; one clade was lethal by intranasal but with 2 of 4 mice surviving intragastric exposure; and one was lethal by 9 days after intranasal exposure and by 10 days after intragastric exposure. Where pathogenicity was demonstrated in mice, respiratory or systemic disease developed, with at most indirect evidence of the presence of viral antigen in intestinal tissues.

1.4.8. Summary for Mouse Studies

Overall, as for previous *in vivo* studies listed in Table 1, mouse studies included few animals per treatment group that may limit robustness of conclusions. Three research groups inoculated both

ferrets and mice intranasally (Eisfeld et al. 2024; Maemura et al. 2023; Lipatov et al. 2009), and mice appear exquisitely more susceptible to H5N1 than ferrets. LD_{50} doses for mice ranged from 2.2 -48 pfu for three H5N1 strains, while a single high dose of 10^6 pfu to ferrets was lethal for all 3 animals for each H5N1 strain tested (LD_{50} for ferrets not estimated)(Maemura et al. 2023). Another study reported a comparable intranasal LD_{50} of 31.6 pfu in mice inocuated with the cow-H5N1 strain (Eisfeld et al. 2024). Asymptomatic infection, observed recently in human cases, was not observed in mice. Thus, the mouse model be unrepresentative of the infectivity and virulence of H5N1 in humans, offering limited utility for extrapolating to natural human exposures.

However, some mouse studies on mechanisms of infection and resistance may be relevant for extrapolation to human systems, including the following: i) pathogenicity was variable by intragastric inoculation, but respiratory or systemic infection was consistently observed, with lack of definitive determination of infection and replication of GI tissues and cells (Lipatov et al. 2009); and ii) the immunomodulating peptide lactoferrin reduced or repaired influenza-induced inflammatory pathology in lung and colon tissues via the gut-lung axis, involving complex interactions with the mucosal immune system, the gut microbiota, inflammatory factors, and the intestinal barrier (Huang et al. 2023).

It appears that Guan and colleagues (Guan et al. 2024) mischaracterized their inoculation intended as oral exposure route to test for GI infection and replication in mice. The presence of inoculum in the nasal cavity, respiratory pathology, and lack of detection in intestine or feces do not support the authors' conclusion that influenza A H5N1 is transmitted by ingestion.

Further, intestinal damage in mice following primary respiratory infection via intranasal inoculation (Huang et al. 2023) was associated with indirect T-cell mediated intestinal immune damage and disruption of microbiota, not GI infection. The functional roles of the gut-lung axis in influenza A H5N1 and in particular lactoferrin were demonstrated in this study, specifically that the dietary immunomodulator lactoferrin reduced lung and intestinal injury, alleviated inflammation, rebalanced gut microbiota, and restored integrity of intestinal wall and lung tissue (Huang et al. 2023). Another *in vivo* mouse study dosed C57BL/6 mice by the intranasal route at one sublethal doses of influenza A H1N1 and demonstrated protection against influenza for mice orally administered the bacterium *Lactobacillus paracasei* by activating interferon signaling, increasing fatty acid production, and enhancing immune response via the gut-lung axis (Kim et al. 2023)

1.5. Domestic Cat Studies

Three *in vivo* studies conducted with domestic cats summarized in Table 1 provide evidence for infectivity and replication for oculo-nasalpharyngeal, intratracheal, intravenous, GI, and direct contact exposures, and two cat studies provide evidence on tissue tropism.

Key findings include the following.

1.5.1. Study 1

Six of 7 anesthetized specific pathogen-free cats inoculated intragastrically in capsules filled with infected chicken liver homogenate at a very high H5N1 dose ($10^{7.8}$ x tissue culture ID_{50}) developed severe systemic infection, and the 7th cat remained swab- and tissue-negative (Reperant et al. 2012). Clinical signs reported were lethargy and reduced appetite. Authors reported dosing two additional cats that were excluded when pharynx was contaminated just after inoculation. Initial cellular tropism was observed in endothelial cells and mononuclear cells in the lamina propria and Peyer's patches of the ileum. Titers were highest in serum samples and nasal swabs, and lower in pharyngeal and rectal swabs by days 3-5. Highest tissue titers were reported in liver and lung. High titers were reported in lymphoid tissues, other respiratory tissues, neural, cardiovascular, and urinary system tissues, and in the esophagus and other GI tissues at days 3-5. Authors noted that dissemination from intragastric inoculation was likely via blood and lymphatic systems, not via neuronal transmission. Widespread hemorrhages were observed in multiple organs, resembling pathogenesis in chickens. Endothelial cells were positive by histopathology in each tissue system analyzed.

1.5.2. Study 2

Two or 4 specific pathogen-free cats per treatment group were inoculated with a very high H5N1 dose (10^{6-7} x egg ID_{50}) in swallowed capsules (oral) or via the oculo-nasopharyngeal route or intravenously (Vahlenkamp et al. 2010). Clinical signs were fever, pharyngeal shedding, depression, anorexia, and labored breathing from 2 days post-inoculation onwards. All inoculated cats developed bronchopneumonia, viremia, and systemic infection, irrespective of route, with titers highest in lung and liver and lowest in colon and jejunum. Irrespective of inoculation route, lung and liver were the main targets of infection and replication. Authors reported little evidence of infection and replication in intestinal tissues and inconsistent detection in rectal swabs, with no apparent lesions and low or negative viral titers in GI tissues.

1.5.3. Study 3

Nine specific pathogen-free cats were inoculated by oculo-nasalpharyngeal route with a dose series (2-3/dose) at 1, 10^2, 10^4, or 10^6 x egg ID_{50} . No disease transmission was observed in cats inoculated at the three lower doses, while three of four cats at the highest dose died.

Ten cats treated with H5N6 vaccine were subsequently inoculated (five each for H5N1 and H5N6) at a high viral dose (10^6 x egg ID_{50}) 4 weeks postvaccination. Vaccinated cats were cross-protected against H5N1.

1.5.4. Study 4

A total of 7 specific pathogen-free European shorthair cats were exposed to H5N1 via intratrachael inoculation (3 tracheally-inoculated donor cats), via co-housing of naïve cats with donors cats (2 naïve recipient cats), and via feeding of inoculated chicks (3 orally-inoculated cats) (Rimmelzwaan et al. 2006). Clinical signs included fever, decreased activity, protrusion of third eyelid, conjunctivitis, and labored breathing. Inoculated and recipient cats in direct contact with inoculated cats developed developed severe respiratory and systemic infections as well as dissemination to nervous, cardiovascular, digestive, lymphoid, endocrine, and urinary systems associated with necrosis and inflammation. Viral antigen expression was highest in lung, adrenal gland, liver, brain, and heart. In lung tissues, cellular tropism was reported for type 1 and type 2 pneumocytes, bornchiolar and bronchial epithelial cells, alveolar macrophages, and rarely endothlial cells. Viral antigen was not detected in GI tissues of all 3 orally-inoculated cats, and not in any cat for esophagus, stomach, pancreas, jejumum, cecum, colon, and urinary bladder. Authors reported that orally inoculated cats may have received inoculum via the respiratory tract in addition to exposure in the GI tract, and that no immunochemical evidence of replication in the epithelium was observed for seven levels of the GI tract sampled.

Three donor cats intratracheally infected with a high H5N1 dose (2.5 x 10^4 tissue culture ID_{50}) transmitted infection to two co-housed naïve recipient cats that showed similar clinical signs as donor cats. All cats developed respiratory and systemic infections with gross pulmonary lesions and histological lesions in lung, adrenal gland, liver, brain, and heart. Nasal and pharyngeal swabs were positive except for one negative recipient cat. Rectal swab titers were variable.

Donor cats had variable tissue titer results for the 17 tissues analyzed (stomach, liver, kidney, heart, cerebrum, cerebellum, brain stem, olfactory bulb, nasal concha, lung, trachea, jejunum, tonsil, eyelid, tracheobronchael lymph node, mesenteric lymph node, and spleen). One donor cat had high titers for all

tissues, the second cat had high titers for kidney and brain with some other tissues low titer, and the third donor cat had high titers for kidney, cerebrum, brain stem, nasal concha, lung, trachea. The two recipient cats had positive tissue titers only for lung and trachea, no detectable titers for the other 15 tissues tested.

Three cats were fed chicks one day after the chicks were inoculated intratracheally with a high H5N1 dose (2.5 x 10^4 tissue culture ID_{50}). Although actual ingested oral doses were not reported for the cats fed inoculated chicks, doses were likely high due to high titers reported in chick liver and lung homogenates (>10^9 tissue culture ID_{50}/g tissue). Orally exposed cats showed similar clinical signs, positive swabs, and respiratory and systemic infection with pulmonary lesions and hemmorhages in tonsils, mandibular and retropharyngeal lymph nodes, and liver for some cats. Histological lesions detected in all three orally inoculated cats included lung, brain, heart, kidney, liver and adrenal gland as per intranasally inoculated cats, as well as small intestinal lesions in two of 3 orally inoculated cats.

Rectal swab titers were lower in orally inoculated cats than the highest intratrachael inoculated cat, suggesting that the virus may not be relplicating in the GI tract. As noted for the intratracheally inoculated cats, tissue titers were variable for three orally inoculated cats. One cat had very high titers for lung, trachea, and brain stem and non-detectable titers in all other tissues; the second cat had moderately high titers for liver, kidney, heart, cerebrum, cerebellum, brain stem, olfactory bulb, nasal concha, lung, trachea, and tonsil and nondetectable otherwise; and the third cat had moderate titers for brain stem, nasal concha, and lung and low to non-detectable titers for other tissues.

1.5.1. Summary for Domestic Cat Studies

Overall, the three cat studies included few animals per treatment group and predominantly very high lethal dose treatments that offer limited utility for extrapolating to natural human exposures. Studies published more recently than these three studies from 2006, 2010, and 2012 were not identified in our searches of the literature. The three available studies provide consistent evidence on clinical signs (fever, decreased activity/lethargy, anorexia/reduced appetite, pharyngeal shedding, protrusion of third eyelid, conjunctivitis, and labored breathing), irrespective of transmission route. Cats typically developed pneumonia, viremia, and systemic infections disseminating to major organ systems. Similarities in lesions and presence of viral antigen were noted in lung (broncho-pneumonia) and liver for all studies, with one or two studies also noting histological lesions in adrenal gland, brain, heart and kidney. Due in part to very small numbers of cats per treatment group, variability within each study was

high, particularly for rectal swab and blood sampling. Some conflicting evidence was reported for cat inoculation studies: Reperant and colleagues reported tropism to endothelial cells as the viral target cell; and Rimmelzwaan and colleagues reported tropism primarily to epithelial cells (Reperant et al. 2012; Rimmelzwaan et al. 2006). Also, uncertainties are noted in these studies regarding infection and replication of H5N1 in GI cells. Overall, the three inoculation studies in cats demonstrate that high H5N1 doses via all routes tested caused respiratory and systemic pathology in cats, though the extent of infection and replication in GI tissues remains uncertain.

1.6. Dog and Cat Study

An *in vivo* study provided evidence for transient fever and positve pharyngeal swabs with some seroconversion in beagle dogs inoculated oculo-nasalpharyngeally with a high dose of H5N1 (10^6 x 50% egg ID; dose in pfu not reported) (Giese et al. 2008). Rectal swabs were negative for inoculated dogs. However, all 4 inoculated dogs recovered, and no transmission was observed to 3 naïve cats and a naïve dog co-housed in direct contact with inoculated donor dogs.

The study authors also summarized partial results of a subsequent experiment inoculating 3 domestic cats with the same high oculo-nasalpharyngeal dose. This dose was fatal to cats that showed severe signs including high fever, decrased activity, conjunctivitis, and labored breathing, with pharyngeal and rectal swabs positive for viral antigen. Hoever, no transmission was observed to naïve dogs cohoused with infected cats. No further details on pathology was provided for cats.

References Cited in Supplemental Materials

Baker, Amy L. 2024. “Experimental Reproduction of Viral Replication and Disease in Dairy Calves and Lactating Cows Inoculated with Highly Pathogenic Avian Influenza H5N1 Clade 2.3.4.4b.” https://www.biorxiv.org/content/10.1101/2024.07.12.603337v1.

Baker, Amy L., Bailey Arruda, Mitchell V. Palmer, Paola Boggiatto, Kaitlyn Sarlo Davila, Alexandra Buckley, Giovana Ciacci Zanella, et al. 2024. “Dairy Cows Inoculated with Highly Pathogenic Avian Influenza Virus H5N1.” *Nature*, October, 1–3. https://doi.org/10.1038/s41586-024-08166-6.

Baskin, Carole R., Helle Bielefeldt-Ohmann, Terrence M. Tumpey, Patrick J. Sabourin, James P. Long, Adolfo García-Sastre, Airn-E. Tolnay, et al. 2009. “Early and Sustained Innate Immune Response Defines Pathology and Death in Nonhuman Primates Infected by Highly Pathogenic Influenza Virus.” *Proceedings of the National Academy of Sciences of the United States of America* 106 (9): 3455–60. https://doi.org/10.1073/pnas.0813234106.

Belser, Jessica A., Kortney M. Gustin, Jacqueline M. Katz, Taronna R. Maines, and Terrence M. Tumpey. 2015. “Comparison of Traditional Intranasal and Aerosol Inhalation Inoculation of Mice with Influenza A Viruses.” *Virology* 481 (July):107–12. https://doi.org/10.1016/j.virol.2015.02.041.

Belser, Jessica A., Kortney M. Gustin, Taronna R. Maines, Mary J. Pantin-Jackwood, Jacqueline M. Katz, and Terrence M. Tumpey. 2012. “Influenza Virus Respiratory Infection and Transmission Following Ocular Inoculation in Ferrets.” *PLOS Pathogens* 8 (3): e1002569. https://doi.org/10.1371/journal.ppat.1002569.

Belser, Jessica A., Xiangjie Sun, Joanna A. Pulit-Penaloza, and Taronna R. Maines. 2024. “Fatal Infection in Ferrets after Ocular Inoculation with Highly Pathogenic Avian Influenza A(H5N1) Virus.” *Emerging Infectious Diseases* 30 (7): 1484–87. https://doi.org/10.3201/eid3007.240520.

Bertran, Kateri, Roser Dolz, and Natàlia Majó. 2014. “Pathobiology of Avian Influenza Virus Infection in Minor Gallinaceous Species: A Review.” *Avian Pathology* 43 (1): 9–25. https://doi.org/10.1080/03079457.2013.876529.

Bertran, Kateri, and David E. Swayne. 2014. “High Doses of Highly Pathogenic Avian Influenza Virus in Chicken Meat Are Required to Infect Ferrets.” *Veterinary Research* 45 (1): 60. https://doi.org/10.1186/1297-9716-45-60.

Buey, Berta, Elena Layunta, Eva Latorre, and Jose Emilio Mesonero. 2023. “Potential Role of Milk Bioactive Peptides on the Serotonergic System and the Gut-Brain Axis.” *International Dairy Journal* 137 (February):105534. https://doi.org/10.1016/j.idairyj.2022.105534.

Burrough, Eric R., Drew R. Magstadt, Barbara Petersen, Simon J. Timmermans, Phillip C. Gauger, Jianqiang Zhang, Chris Siepker, et al. 2024. “Highly Pathogenic Avian Influenza A(H5N1) Clade 2.3.4.4b Virus Infection in Domestic Dairy Cattle and Cats, United States, 2024.” *Emerging Infectious Diseases* 30 (7). https://doi.org/10.3201/eid3007.240508.

“CDC Newsroom.” 2024. CDC. July 3, 2024. https://www.cdc.gov/media/releases/2024/p-0703-4th-human-case-h5.html.

Cillóniz, Cristian, Kyoko Shinya, Xinxia Peng, Marcus J. Korth, Sean C. Proll, Lauri D. Aicher, Victoria S. Carter, et al. 2009. “Lethal Influenza Virus Infection in Macaques Is Associated with Early Dysregulation of Inflammatory Related Genes.” *PLOS Pathogens* 5 (10): e1000604. https://doi.org/10.1371/journal.ppat.1000604.

Corry, Jacqueline, Gwenddolen Kettenburg, Amit A. Upadhyay, Megan Wallace, Michelle M. Marti, Elizabeth R. Wonderlich, Stephanie J. Bissel, et al. 2022. “Infiltration of Inflammatory Macrophages and Neutrophils and Widespread Pyroptosis in Lung Drive Influenza Lethality in Nonhuman Primates.” *PLoS Pathogens* 18 (3): e1010395. https://doi.org/10.1371/journal.ppat.1010395.

Eisfeld, Amie J., Asim Biswas, Lizheng Guan, Chunyang Gu, Tadashi Maemura, Sanja Trifkovic, Tong Wang, et al. 2024. “Pathogenicity and Transmissibility of Bovine H5N1 Influenza Virus.” *Nature*, July, 1–3. https://doi.org/10.1038/s41586-024-07766-6.

Gallo, Valentina, Alyexandra Arienzo, Federica Tomassetti, and Giovanni Antonini. 2024. “Milk Bioactive Compounds and Gut Microbiota Modulation: The Role of Whey Proteins and Milk Oligosaccharides.” *Foods* 13 (6): 907. https://doi.org/10.3390/foods13060907.

Giese, Matthias, Timm C. Harder, Jens P. Teifke, Robert Klopfleisch, Angele Breithaupt, Thomas C. Mettenleiter, and Thomas W. Vahlenkamp. 2008. “Experimental Infection and Natural Contact Exposure of Dogs with Avian Influenza Virus (H5N1).” *Emerging Infectious Diseases* 14 (2): 308–10. https://doi.org/10.3201/eid1402.070864.

Guan, Lizheng, Amie J. Eisfeld, David Pattinson, Chunyang Gu, Asim Biswas, Tadashi Maemura, Sanja Trifkovic, et al. 2024. “Cow’s Milk Containing Avian Influenza A(H5N1) Virus - Heat Inactivation and Infectivity in Mice.” *The New England Journal of Medicine*, May. https://doi.org/10.1056/NEJMc2405495.

Huang, Yanyi, Peiyang Zhang, Shuyi Han, and Hongxuan He. 2023. “Lactoferrin Alleviates Inflammation and Regulates Gut Microbiota Composition in H5N1-Infected Mice.” *Nutrients* 15 (15): 3362. https://doi.org/10.3390/nu15153362.

Kalthoff, Donata, Bernd Hoffmann, Timm Harder, Markus Durban, and Martin Beer. 2008. “Experimental Infection of Cattle with Highly Pathogenic Avian Influenza Virus (H5N1).” *Emerging Infectious Diseases* 14 (7): 1132. https://doi.org/10.3201/eid1407.071468.

Kanekiyo, Masaru, Rebecca A. Gillespie, Morgan Midgett, Katherine J. O’Malley, Connor Williams, Syed M. Moin, Megan Wallace, et al. 2023. “Refined Semi-Lethal Aerosol H5N1 Influenza Model in Cynomolgus Macaques for Evaluation of Medical Countermeasures.” *iScience* 26 (10). https://doi.org/10.1016/j.isci.2023.107830.

Kim, Seungil, Sohyeon Lee, Tae-Young Kim, Su-Hyun Lee, Sang-Uk Seo, and Mi-Na Kweon. 2023. “Newly Isolated Lactobacillus Paracasei Strain Modulates Lung Immunity and Improves the Capacity to Cope with Influenza Virus Infection.” *Microbiome* 11 (November):260. https://doi.org/10.1186/s40168-023-01687-8.

Lipatov, Aleksandr S., Yong Kuk Kwon, Mary J. Pantin-Jackwood, and David E. Swayne. 2009. “Pathogenesis of H5N1 Influenza Virus Infections in Mice and Ferret Models Differs According to Respiratory Tract or Digestive System Exposure.” *The Journal of Infectious Diseases* 199 (5): 717–25. https://doi.org/10.1086/596740.

Lockhart, Ainsley, Daniel Mucida, and Roham Parsa. 2022. “Immunity to Enteric Viruses.” *Immunity* 55 (5): 800–818. https://doi.org/10.1016/j.immuni.2022.04.007.

Maemura, Tadashi, Lizheng Guan, Chunyang Gu, Amie Eisfeld, Asim Biswas, Peter Halfmann, Gabriele Neumann, and Yoshihiro Kawaoka. 2023. “Characterization of Highly Pathogenic Clade 2.3.4.4b H5N1 Mink Influenza Viruses.” *eBioMedicine* 97 (November). https://doi.org/10.1016/j.ebiom.2023.104827.

McAfee, Aaron. 2024. “Raw Farm Weekly Raw Dairy Production for California Retail Market from 2019-2024 and H5N1 PCR Testing Results for 2024.”

Michaelis, Martin, Janina Geiler, Denise Klassert, Hans Wilhelm Doerr, and Jindrich Cinatl Jr. 2009. “Infection of Human Retinal Pigment Epithelial Cells with Influenza A Viruses.” *Investigative Ophthalmology & Visual Science* 50 (11): 5419–25. https://doi.org/10.1167/iovs.09-3752.

Mims, C. A. 1989. “The Pathogenetic Basis of Viral Tropism.” *The American Journal of Pathology* 135 (3): 447–55.

Mooij, Petra, Marieke A. Stammes, Daniella Mortier, Zahra Fagrouch, Nikki van Driel, Ernst J. Verschoor, Ivanela Kondova, Willy M. J. M. Bogers, and Gerrit Koopman. 2021. “Aerosolized Exposure to H5N1 Influenza Virus Causes Less Severe Disease Than Infection via Combined Intrabronchial, Oral, and Nasal Inoculation in Cynomolgus Macaques.” *Viruses* 13 (2): 345. https://doi.org/10.3390/v13020345.

Muramoto, Yukiko, Jason E. Shoemaker, Mai Quynh Le, Yasushi Itoh, Daisuke Tamura, Yuko Sakai-Tagawa, Hirotaka Imai, et al. 2014. "Disease Severity Is Associated with Differential Gene Expression at the Early and Late Phases of Infection in Nonhuman Primates Infected with Different H5N1 Highly Pathogenic Avian Influenza Viruses." *Journal of Virology* 88 (16): 8981–97. https://doi.org/10.1128/jvi.00907-14.

Ng, Tzi Bun, Randy Chi Fai Cheung, Jack Ho Wong, Yan Wang, Denis Tsz Ming Ip, David Chi Cheong Wan, and Jiang Xia. 2015. "Antiviral Activities of Whey Proteins." *Applied Microbiology and Biotechnology* 99 (17): 6997–7008. https://doi.org/10.1007/s00253-015-6818-4.

Nicas, Mark, and Rachael M. Jones. 2009. "Relative Contributions of Four Exposure Pathways to Influenza Infection Risk." *Risk Analysis* 29 (9): 1292–1303. https://doi.org/10.1111/j.1539-6924.2009.01253.x.

Nolan, L.S., J.M. Rimer, and M. Good. 2020. "The Role of Human Milk Oligosaccharides and Probiotics on the Neonatal Microbiome and Risk of Necrotizing Enterocolitis." *A Narrative Review. Nutrients* 12 (10): 3052. https://doi.org/10.3390/nu12103052.

Oliver, Isabel, Jonathan Roberts, Colin S. Brown, Alexander Mp Byrne, Dominic Mellon, Rowena DE Hansen, Ashley C. Banyard, et al. 2022. "A Case of Avian Influenza A(H5N1) in England, January 2022." *Euro Surveillance: Bulletin Europeen Sur Les Maladies Transmissibles = European Communicable Disease Bulletin* 27 (5): 2200061. https://doi.org/10.2807/1560-7917.ES.2022.27.5.2200061.

Pulit-Penaloza, Joanna A., Nicole Brock, Jessica A. Belser, Xiangjie Sun, Claudia Pappas, Troy J. Kieran, Poulami Basu Thakur, et al. 2024. "Highly Pathogenic Avian Influenza A(H5N1) Virus of Clade 2.3.4.4b Isolated from a Human Case in Chile Causes Fatal Disease and Transmits between Co-Housed Ferrets." *Emerging Microbes & Infections* 0 (ja): 2332667. https://doi.org/10.1080/22221751.2024.2332667.

"QMRAwiki Influenza Dose Response for Intranasal." n.d. Accessed June 25, 2024. https://qmrawiki.org/pathogens/influenza.

Reperant, Leslie A., Marco W. G. van de Bildt, Geert van Amerongen, Lonneke M. E. Leijten, Simon Watson, Anne Palser, Paul Kellam, et al. 2012. "Marked Endotheliotropism of Highly Pathogenic Avian Influenza Virus H5N1 Following Intestinal Inoculation in Cats." *Journal of Virology* 86 (2): 1158–65. https://doi.org/10.1128/jvi.06375-11.

Restori, Katherine H., Kayla M. Septer, Cassandra J. Field, Devanshi R. Patel, David VanInsberghe, Vedhika Raghunathan, Anice C. Lowen, and Troy C. Sutton. 2024. "Risk Assessment of a Highly Pathogenic H5N1 Influenza Virus from Mink." *Nature Communications* 15 (1): 4112. https://doi.org/10.1038/s41467-024-48475-y.

Richard, Mathilde, Judith M. A. van den Brand, Theo M. Bestebroer, Pascal Lexmond, Dennis de Meulder, Ron A. M. Fouchier, Anice C. Lowen, and Sander Herfst. 2020. "Influenza A Viruses Are Transmitted via the Air from the Nasal Respiratory Epithelium of Ferrets." *Nature Communications* 11 (1): 766. https://doi.org/10.1038/s41467-020-14626-0.

Rimmelzwaan, G. F., T. Kuiken, G. van Amerongen, T. M. Bestebroer, R. A. M. Fouchier, and A. D. M. E. Osterhaus. 2001. "Pathogenesis of Influenza A (H5N1) Virus Infection in a Primate Model." *Journal of Virology* 75 (14): 6687–91. https://doi.org/10.1128/jvi.75.14.6687-6691.2001.

Rimmelzwaan, G. F., Debby van Riel, Marianne Baars, Theo M. Bestebroer, Geert van Amerongen, Ron A.M. Fouchier, Albert D.M.E. Osterhaus, and Thijs Kuiken. 2006. "Influenza A Virus (H5N1) Infection in Cats Causes Systemic Disease with Potential Novel Routes of Virus Spread within and between Hosts." *The American Journal of Pathology* 168 (1): 176–83.

Rosenke, Kyle, Heinz Feldmann, Amanda Griffin, Franziska Kaiser, Ekaterina Altynova, Reshma Mukesh, Meaghan Flagg, et al. 2024. "Orogastric Exposure of Cynomolgus Macaques to Bovine HPAI H5N1 Virus Results in Subclinical Infection." https://doi.org/10.21203/rs.3.rs-5182487/v1.

Ruan, Tao, Yuling Sun, Jingting Zhang, Jing Sun, Wei Liu, Richard A. Prinz, Daxin Peng, Xiufan Liu, and Xiulong Xu. 2022. "H5N1 Infection Impairs the Alveolar Epithelial Barrier through Intercellular

Junction Proteins via Itch-Mediated Proteasomal Degradation." *Communications Biology* 5 (1): 186. https://doi.org/10.1038/s42003-022-03131-3.

Santos, I, M Silva, M Gracio, L Pedroso, and A Lima. 2024. "Milk Antiviral Proteins and Derived Peptides against Zoonoses." 2024. https://www.mdpi.com/1422-0067/25/3/1842.

Shu, Yuelong, Chris Ka-fai Li, Zi Li, Rongbao Gao, Qian Liang, Ye Zhang, Libo Dong, et al. 2010. "Avian Influenza A(H5N1) Viruses Can Directly Infect and Replicate in Human Gut Tissues." *The Journal of Infectious Diseases* 201 (8): 1173–77. https://doi.org/10.1086/651457.

Szablewski, Christine M., Chelsea Iwamoto, Sonja J. Olsen, Carolyn M. Greene, Lindsey M. Duca, C. Todd Davis, Kira C. Coggeshall, et al. 2023. "Reported Global Avian Influenza Detections Among Humans and Animals During 2013-2022: Comprehensive Review and Analysis of Available Surveillance Data." *JMIR Public Health and Surveillance* 9 (August):e46383. https://doi.org/10.2196/46383.

Vahlenkamp, Thomas W., Timm C. Harder, Matthias Giese, Fengsheng Lin, Jens P. Teifke, Robert Klopfleisch, Ralf Hoffmann, Ian Tarpey, Martin Beer, and Thomas C. Mettenleiter. 2008. "Protection of Cats against Lethal Influenza H5N1 Challenge Infection." *Journal of General Virology* 89 (4): 968–74. https://doi.org/10.1099/vir.0.83552-0.

Vahlenkamp, Thomas W., Jens P. Teifke, Timm C. Harder, Martin Beer, and Thomas C. Mettenleiter. 2010. "Systemic Influenza Virus H5N1 Infection in Cats after Gastrointestinal Exposure." *Influenza and Other Respiratory Viruses* 4 (6): 379–86. https://doi.org/10.1111/j.1750-2659.2010.00173.x.

Wang, Yi-Hsiang, Aniket Limaye, Je-Ruei Liu, and Tai-Na Wu. 2023. "Potential Probiotics for Regulation of the Gut-Lung Axis to Prevent or Alleviate Influenza in Vulnerable Populations." *Journal of Traditional and Complementary Medicine* 13 (2): 161–69. https://doi.org/10.1016/j.jtcme.2022.08.004.

Watanabe, Tokiko, Kiyoko Iwatsuki-Horimoto, Maki Kiso, Noriko Nakajima, Kenta Takahashi, Tiago Jose da Silva Lopes, Mutsumi Ito, Satoshi Fukuyama, Hideki Hasegawa, and Yoshihiro Kawaoka. 2018. "Experimental Infection of Cynomolgus Macaques with Highly Pathogenic H5N1 Influenza Virus through the Aerosol Route." *Scientific Reports* 8 (1): 4801. https://doi.org/10.1038/s41598-018-23022-0.

Wonderlich, Elizabeth R., Zachary D. Swan, Stephanie J. Bissel, Amy L. Hartman, Jonathan P. Carney, Katherine J. O'Malley, Adebimpe O. Obadan, et al. 2017. "Widespread Virus Replication in Alveoli Drives Acute Respiratory Distress Syndrome in Aerosolized H5N1 Influenza Infection of Macaques." *Journal of Immunology (Baltimore, Md.: 1950)* 198 (4): 1616–26. https://doi.org/10.4049/jimmunol.1601770.

Yu, Wendy C. L., Renee W. Y. Chan, Jieru Wang, Emily A. Travanty, John M. Nicholls, J. S. Malik Peiris, Robert J. Mason, and Michael C. W. Chan. 2011. "Viral Replication and Innate Host Responses in Primary Human Alveolar Epithelial Cells and Alveolar Macrophages Infected with Influenza H5N1 and H1N1 Viruses." *Journal of Virology* 85 (14): 6844–55. https://doi.org/10.1128/JVI.02200-10.

Zeng, Hui, Cynthia S. Goldsmith, Amrita Kumar, Jessica A. Belser, Xiangjie Sun, Claudia Pappas, Nicole Brock, et al. 2019. "Tropism and Infectivity of a Seasonal A(H1N1) and a Highly Pathogenic Avian A(H5N1) Influenza Virus in Primary Differentiated Ferret Nasal Epithelial Cell Cultures." *Journal of Virology* 93 (10): e00080-19. https://doi.org/10.1128/JVI.00080-19.

Zhao, Na, Supen Wang, Lan Wang, Yingying Shi, Yixin Jiang, Tzu-Jung Tseng, Shelan Liu, Ta-Chien Chan, and Zhiruo Zhang. 2023. "Epidemiological Features and Trends in the Mortality Rates of 10 Notifiable Respiratory Infectious Diseases in China from 2004 to 2020: Based on National Surveillance." *Frontiers in Public Health* 11:1102747. https://doi.org/10.3389/fpubh.2023.1102747.

Zimecki, Michał, Jeffrey K. Actor, and Marian L. Kruzel. 2021. "The Potential for Lactoferrin to Reduce SARS-CoV-2 Induced Cytokine Storm." *International Immunopharmacology* 95 (June):107571. https://doi.org/10.1016/j.intimp.2021.107571.
